# Karyotypic Determinants of Chromosome Instability in Aneuploid Budding Yeast

**DOI:** 10.1371/journal.pgen.1002719

**Published:** 2012-05-17

**Authors:** Jin Zhu, Norman Pavelka, William D. Bradford, Giulia Rancati, Rong Li

**Affiliations:** 1 Stowers Institute for Medical Research, Kansas City, Missouri, United States of America; 2 Singapore Immunology Network Agency of Science, Technology, and Research, Singapore, Singapore; 3 Institute of Medical Biology, Agency of Science, Technology, and Research, Singapore, Singapore; 4 Department of Molecular and Integrative Physiology, University of Kansas Medical Center, Kansas City, Kansas, United States of America; Duke University, United States of America

## Abstract

Recent studies in cancer cells and budding yeast demonstrated that aneuploidy, the state of having abnormal chromosome numbers, correlates with elevated chromosome instability (CIN), i.e. the propensity of gaining and losing chromosomes at a high frequency. Here we have investigated ploidy- and chromosome-specific determinants underlying aneuploidy-induced CIN by observing karyotype dynamics in fully isogenic aneuploid yeast strains with ploidies between 1N and 2N obtained through a random meiotic process. The aneuploid strains exhibited various levels of whole-chromosome instability (i.e. chromosome gains and losses). CIN correlates with cellular ploidy in an unexpected way: cells with a chromosomal content close to the haploid state are significantly more stable than cells displaying an apparent ploidy between 1.5 and 2N. We propose that the capacity for accurate chromosome segregation by the mitotic system does not scale continuously with an increasing number of chromosomes, but may occur via discrete steps each time a full set of chromosomes is added to the genome. On top of such general ploidy-related effect, CIN is also associated with the presence of specific aneuploid chromosomes as well as dosage imbalance between specific chromosome pairs. Our findings potentially help reconcile the divide between gene-centric versus genome-centric theories in cancer evolution.

## Author Summary

Aneuploidy, the state of harboring an unbalanced number of chromosomes, has long been hypothesized to be at the basis of malignant transformation. Recent studies have also shown that aneuploidy is an important form of genome alteration underlying adaptive evolution of cells in response to harsh environments or genetic perturbations. In addition to the profound effect that aneuploidy has on gene expression and phenotype, another feature thought to contribute to aneuploidy's role in cancer and cellular evolution is the heightened chromosome instability of aneuploid cells. Since chromosome instability is the condition of gaining and losing chromosomes at a high frequency, this could lead to a vicious cycle in which aneuploidy could lead to further enhanced genetic diversity. Given the ever-changing and heterogeneous aneuploid cell populations, and the difficulty of separating the effect of aneuploidy from other types of genetic aberrations, the molecular mechanisms underlying aneuploidy-driven chromosome instability have remained largely unexplored. Here we describe the first unbiased and systematic investigation of chromosome instability associated with aneuploid genomes in the budding yeast *Saccharomyces cerevisiae*. Our results revealed both genome-level and chromosome-specific determinants of chromosome instability in aneuploid yeast. Our findings potentially help explain the molecular mechanism underlying a major source of genome instability in cancer.

## Introduction

The nature of the genetic changes driving cellular evolution has been a central issue in both adaptive evolution of unicellular organisms and somatic evolution of cancer cells. Phenotypic variation, acting as a substrate of Darwinian selection and as an origin of phenotypic innovation, can be driven by sequence-based mutations as well as copy number changes [1], [2], [3], [4], [5], [6], [7]. In cancer, the gene-centric theory posits that cancer progression is driven by sequence alterations in specific genes playing key roles in cell cycle control and genome stability, leading to malignant growth and accumulation of further genetic aberrations [8]. Under this perspective, aneuploidy is more likely to be an innocent byproduct than a driver of the evolutionary process leading towards malignant transformation. The chromosome theory, on the other hand, emphasizes the cytogenetic diversity in cancer and proposes that it is the abnormal chromosome copy numbers, or aneuploidy, rather than variation in specific gene sequences, that accounts for both the loss of growth control and the remarkable adaptability of tumor cell populations toward restrictive tissue environments or chemotherapy [9]. According to this theory, aneuploidy would lead to increased rates of various types of genomic instability, including chromosome instability (CIN), and therefore a continuous ability to generate new adaptive aneuploid genomes. This potential snowballing effect has been termed “genome chaos” and has been hypothesized to be at the basis of malignant transformation [9], [10], [11]. While the gene-centric theory of cancer is widely accepted, understanding the mechanisms by which aneuploidy could lead to CIN might reconcile the two theories. For example, is the increased CIN in an aneuploid genome a result of the abnormal chromosome numbers *per se* or of aneuploidy-driven alteration of the expression of specific genes?

Whereas studying the contribution of aneuploidy to CIN in cancer cells is complicated by the fact that most cancer cells possess both numerous point mutations and other kinds of chromosome abnormalities [6], [12], [13], simple model organisms such as the yeast *Saccharomyces cerevisiae* represent valuable systems for assessing independent effects of individual types of genetic changes. Budding yeast cells are especially suitable for these types of studies because they tolerate aneuploidy relatively well [14], [15], most likely because their relatively small haploid genome (∼6,000 ORFs over ∼12 million base pairs) is segmented into a relatively large number of chromosomes (N = 16). Several studies have shown that aneuploid yeast cells not only are characterized by phenotypic variation but also exhibit genome instability [15], [16], [17], [18]. For example, two independent studies with congenic aneuploid strains obtained by sporulation of triploid or pentaploid yeast found that, while some of the aneuploid strains were relatively stable, the majority of the strains were chromosomally unstable [15], [17]. Another paper recently reported decreased artificial chromosome transmission fidelity and elevated mitotic recombination in a set of disomic yeast strains compared to the haploid parent [18]. However, none of these studies investigated into the cellular mechanisms by which an aneuploid karyotype causes CIN.

Insights into the mechanisms by which aneuploidy leads to CIN are important for understanding the dynamics of the cellular adaptation process and may ultimately enhance our ability to predict or modulate cancer progression. For example, as stable phenotypes are likely to require a certain degree of genetic stability, there may exist metastable aneuploid constellations amidst the genome chaos. If this was true, what may be the determinants underlying stable or unstable aneuploidy? Formally, aneuploidy could cause CIN through three conceptually distinct though not mutually exclusive mechanisms. First, as aneuploidy leads to varying degrees of growth impairment compared to euploids under standard culture conditions [14], [15], CIN may be induced by the cellular stress present under such conditions. If this hypothesis were correct, then CIN would correlate with the level of growth impairment in specific aneuploid strains. Second, according to the genome chaos theory, the more chromosomes are in aneuploidy in a genome the more unstable that karyotype is expected to be [19]. If this hypothesis were true, then CIN would correlate with how far a strain deviates from the nearest euploid state. Third, it is possible that aneuploid chromosome stoichiometry leads to dosage imbalance for specific genes encoding structural or regulatory components that ensure chromosome stability. This possibility was previously proposed based on imbalance of mitotic spindle components directly involved in chromosome segregation [20]. If this were correct, then correlations might be found between CIN and the relative copy numbers of specific chromosomes or combinations thereof.

In this paper we used an unbiased approach to examine the karyotypic features underlying CIN by generating random aneuploid karyotypes through triploid meiosis and following the dynamics of aneuploid populations with distinct original karyotypes. Our results support a model in which CIN is promoted by both genome-level and chromosome/gene-specific determinants.

## Results

### Aneuploid yeast strains generated from triploid meiosis display varying degrees of chromosome instability

Two methods were instrumental for the analysis explained below in this study. First, we used a high-throughput flow cytometry-based (FACS) assay to determine the overall genome content (referred to as ploidy) of a population of yeast cells. A non-integer ploidy revealed by FACS is likely to correspond to an aneuploid genome. However, FACS data is insufficient to reveal copy number for each chromosome. For this we used a recently established method for determining the relative copy number for each of the 16 yeast chromosomes that is based on quantitative polymerase chain reaction (qPCR) [15]. Combining the ploidy information revealed by FACS and chromosome stoichiometry revealed by qPCR allows determination of an aneuploid karyotype [15].

**Figure 1:**
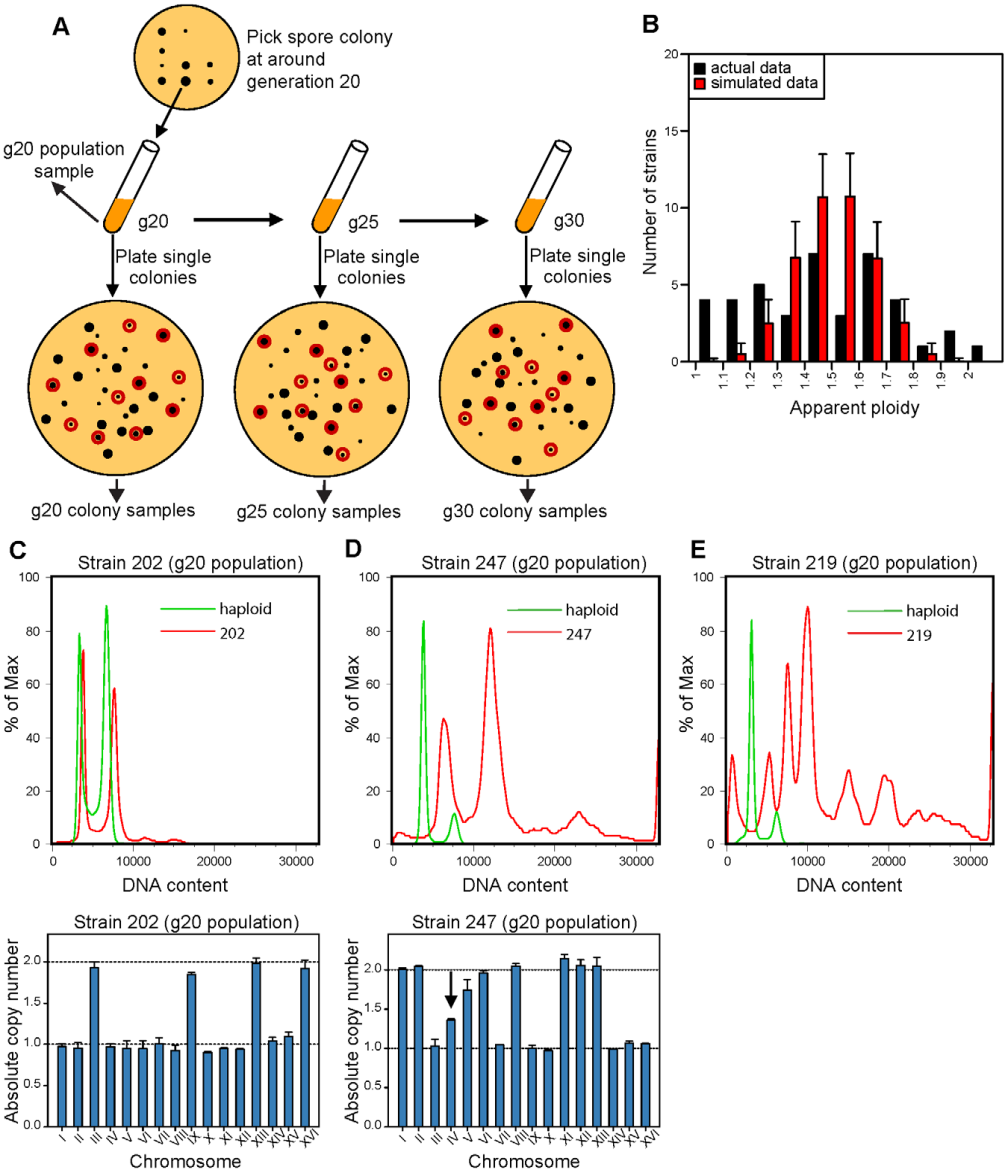
Isolation and karyotype analysis of aneuploid spore colonies after triploid meiosis. (A) Schematic representation of the experimental design used to follow karyotype changes in freshly generated aneuploid strain populations that resulted from triploid meiotic progeny. The g20 population sample was used to determine the original karyotype of the aneuploid spore that seeded the colony. Black dots represent colonies; red circles indicate the 11 randomly chosen colonies resulted from plating the population culture at different generations, which were used to determine the karyotype variation within the population. (B) Distribution of the apparent ploidy based on FACS analysis of the g20 population samples of the collected aneuploid strains as represented in (A). Red bars represent the expected apparent ploidy distribution by simulating random segregation of homologs during meiosis I of the triploid strain. Black bars represent the apparent ploidy distribution obtained experimentally. Error bars represent standard deviations from 10,000 independent simulations. (C–D) Representative DNA content profiles by FACS (upper panels) and chromosome copy numbers by qPCR (lower panels) of two aneuploid strains, for which the original karyotype was directly determined from the g20 population data (C) or indirectly inferred from colony data, i.e. from the most commonly observed copy number across the analyzed colonies (D). Black arrow indicates a chromosome with a non-integer copy number in the population. (E) Representative DNA FACS profile of the g20 population of an aneuploid strain for which the modal ploidy could not be reliably determined and that was hence excluded from further study.

In our previous work [15], we generated isogenic aneuploid yeast strains with random chromosome stoichiometries as meiotic products from sporulated homozygous triploid or pentaploid strains of the S288c background. During meiosis I, chromosome segregation of an odd number of homologs leads to highly frequent aneuploid spore progenies with random karyotypes [16]. In this work, we took a similar approach to generate fully isogenic aneuploid yeast strains, and consistent with previous studies [15], [17], 45% of the aneuploid meiotic products were viable and gave rise to colonies (52 viable spores out of 116 expected from 29 tetrads). Unlike our previous study [15], however, where aneuploid strains with stable karyotypes were chosen for phenotypic comparison and gene expression analyses, in this study our goal was to follow karyotype changes for all (within our experimental limitations, see below) viable aneuploid spores resulting from triploid meiosis. Due to a lack of established methods for single-cell karyotyping in yeast, however, we devised a population-based approach (Figure 1A) that was predicated on the assumption that the modal karyotype of the population within a small colony reflects the karyotype of the cell that seeded the colony.

As illustrated in Figure 1A, the colony grown from each of the 52 viable spores was picked in its entirety after the spore had undergone ∼20 cell divisions, resuspended in liquid media and the actual cell number and thus the number of cell divisions was estimated (see Materials and Methods for details). As the aneuploid colonies grew at different rates, the colonies were picked at different times after tetrad dissection, and the time of colony picking was recorded. Each resulting culture at this time point was called generation 20 (g20) population sample. Due to contamination, only 47 g20 populations were obtained and further analyzed (see Figure S1). A small aliquot of the g20 population sample for each spore colony was used for FACS and qPCR karyotyping analysis, giving rise to the g20 population data (see below). If the initial aneuploid karyotype of a growing spore colony were unstable, karyotype heterogeneity would be expected within the g20 population. This karyotype heterogeneity could in turn allow us to estimate the level of CIN of the initial aneuploid karyotype (see below). To observe it, ∼200 cells from each g20 population sample were spread onto a YPD plate. As soon as the resulting colonies were visible, 11 colonies were randomly chosen from each plate (see Materials and Methods), harvested and frozen for prospective karyotype analysis by FACS and qPCR. These were referred to as the g20 colony samples, and a total of 47×11 = 517 such samples were harvested and stored.

Since some aneuploid karyotypes were more stable than others, to allow for more cell divisions that could give rise to karyotypic deviants, a part of each g20 population sample was further cultured in liquid for 5 and 10 more generations to yield g25 and g30 population samples, respectively, and the time when each of the cultures reached these numbers of generations was recorded. To estimate the number of cell divisions required for detecting karyotypic deviants, we performed computer simulations to calculate the fraction of cells with deviant karyotypes based on different chromosome mis-segregation rates at different generation times. We found that >10% of a cell population is expected to display a deviant karyotype after 20 generations (cell divisions) in the presence of a very high CIN level (1×10^−3^ chromosome mis-segregation per generation) or after 30 generations with a lower CIN level (5×10^−4^ chromosome mis-segregation per generation, Figure S2A and S2B). For a comparison, wild-type diploid and tetraploid yeasts were reported to have an artificial chromosome loss rate of 2.6×10^−4^ and 5.7×10^−2^ per generation, respectively [21]. Again, to determine karyotype diversity within each population, ∼200 cells from each g25 or g30 population were spread onto YPD plates, and 11 colonies from each plate were randomly selected and stored for later karyotyping by FACS and qPCR (Figure 1A). These were referred to as the g25 and g30 colony samples, and a total of 2×47×11 = 1034 such samples were obtained. We did not extend the above procedure beyond generation 30 due to the increasing effect of growth competition on karyotype diversity within each population. Nevertheless, our experimental design was likely to somewhat under-estimate the karyotype diversity for the aneuploid populations as a result of growth competition.

Having obtained and stored away all samples as described above, we first performed FACS analysis on all g20 population samples (Figure S3). One population (strain 221) displayed an apparent ploidy of 2.1 by FACS but subsequent qPCR karyotyping indicated that it was a hypo-diploid. Another g20 aneuploid population (strain 242) had a ploidy over 2N, possibly due to a whole-genome duplication event, and was not included in further analysis (Figure S1). Five of the g20 populations exhibited FACS profiles suggesting an extreme level of DNA content heterogeneity in the population, characterized by the presence of multiple broad peaks with no clear G1 and G2 peaks (Figure 1E and Figure S3). These 5 strains were not included in further analysis due to the difficulty to determine the karyotype makeup of the population (see Figure S1). Conversely, the FACS profiles of the remaining 41 g20 population samples displayed a more homogeneous, albeit aneuploid, DNA content between 1N and 2N with clearly identifiable G1 and G2 peaks (Figure 1B–1D and Figure S3). The ploidy distribution of these 41 populations are more uniform compared to the binomial distribution expected from triploid meiosis (Figure 1B), with significantly fewer than expected viable strains with a ploidy ∼1.5 (Kolmogorov-Smirnov test between observed and expected cumulative distribution function P = 1.58×10^−2^, Figure S4). This result suggests that the viability of aneuploid strains may be biased toward those with ploidy close to a euploid state (haploid or diploid) compared to those with ploidy equidistant to the two nearby euploid states.

We next subjected the above 41 g20 population samples to qPCR karyotyping analysis in order to determine the modal karyotype of each population by combining the ploidy estimate from FACS with the chromosome stoichiometry data from qPCR karyotyping assays. This was successfully accomplished for 36 of the g20 populations where the dominant karyotype could be clearly determined (Figure 1C, showing one such example, and Figure S5, showing all qPCR data for the g20 populations). The remaining 5 g20 populations were simply too heterogeneous in qPCR profiles for us to determine the modal karyotype (Figure S5 “too heterogeneous”). These initial observations already indicate that different aneuploid strains exhibit different levels of CIN.

### Karyotype dynamics in aneuploid populations

In order to associate specific CIN level with specific aneuploid karyotypes, we observed karyotype dynamics in each of the aneuploid strain populations by determining the karyotypes of the 11 randomly selected colonies plated from the population culture at one of the three (g20, g25 and g30) time points (see Figure 1A). Because qPCR karyotyping was of significant cost, only 27 of the 36 strains described above were subjected to this analysis while 9 were excluded due to contamination or redundancy in karyotype with other aneuploid strains in the collection (see Figure S1). We first used FACS data from the colonies to help select the time point most appropriate for karyotype analysis. For those strains that appeared to be most stable (g20 and g25 colonies showing ploidy variation similar to a wild-type control), colonies from the g30 populations were chosen to maximize the chance of observing some karyotypic deviants, whereas for those strains displaying the greatest apparent instability by FACS (colonies showing ploidy variation substantially larger than a wild-type control) colonies of populations from the earlier time points (g20 and g25) were chosen for qPCR analysis. Figure S6 displays representative examples of a haploid control (A), relatively stable (B) and unstable (C) aneuploid strain. The karyotyping data from the g20 population samples and g20, g25 or g30-derived colony samples allowed us to examine the relationship among the observed aneuploid karyotypes of all analyzed samples originated from the same spore.

We next used a haplotype mapping approach to determine the minimum number of chromosome gain or loss events sufficient to explain the diverse karyotypes revealed by the 11 analyzed colonies of a given population. Haplotype maps are typically used to display genetic variation based on SNP loci and help to study the genotypic variation between populations of individuals [22]. A parsimony approach is used during the reconstruction of the relationship between the observed genotypes and do not require *a priori* information regarding the phylogeny of the individuals in the population. In adopting this approach, the 16 yeast chromosomes were treated as independent loci, each of which can exist in, and change between, two states defined by copy numbers (analogous to alleles) – 1 or 2 copies (as the ploidy of these strains varied between 1N and 2N). The algorithm proceeded by attempting to connect all 12 karyotypes (the g20 population karyotype +11 colony karyotypes) in a single haplotype map (in this case, a ‘karyotype map’) by minimizing the total number of mutational events (in this case, copy number changes) in the entire map. It has to be noted that the parsimony approach underlying this algorithm allows distinguishing those colony karyotypes that most likely originated directly from a CIN event in the ancestral spore karyotype from those that arose as secondary events from already deviant karyotypes accumulated in the population. This step was important to not over-estimate the level of CIN that could be attributed to the original spore karyotypes.

**Figure 2:**
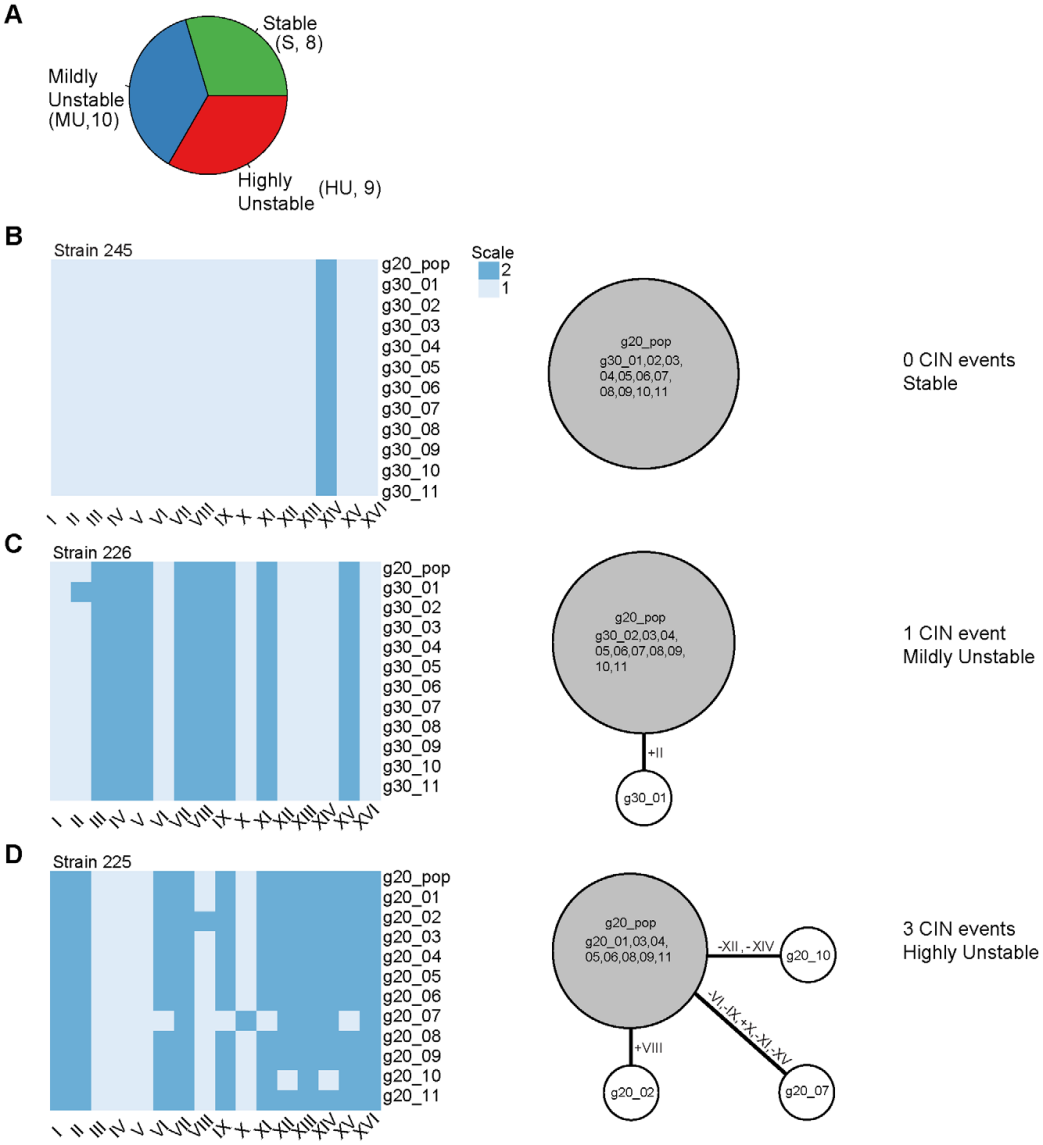
Determination of karyotype changes in aneuploid strain populations. (A) Classification of the CIN level of the 27 analyzed aneuploid strains as stable (S, no CIN event linked to g20 population karyotype), mildly unstable (MU, 1 CIN event linked to g20 population karyotype) or highly unstable (HU, 2 or more CIN events linked to g20 population karyotype). The number of strains belonging to each CIN class is shown in parenthesis. (B–D) Karyotypes of the g20 population sample and of the eleven g30 colonies (left) and the reconstructed karyotype network (right) are shown for a representative S strain (B), MU strain (C) and HU strain (D). For the karyotype network, the area of the circles is proportional to the frequency each karyotype was found among the karyotyped samples (g20 population and g30 colonies); the circle containing the g20 population sample is depicted in gray; white circles represent the karyotypes of the g20 or g30 colonies (11 total) which were divergent from the g20 population karyotype due to loss (minus sign) and/or gain (plus sign) of specific chromosomes (in Roman letters). The labels inside the circles indicate the specific samples whose karyotypes are shown in the heat map on the left. Karyotypic relationship was reconstructed by using a parsimony approach and represented by lines connecting the different karyotypes; thicker connectors refer to the CIN events directly linked to the g20 population karyotype and used for the classification of the strains into CIN categories. The same information for all analyzed aneuploid strains is presented in Figure S7.

**Figure 3:**
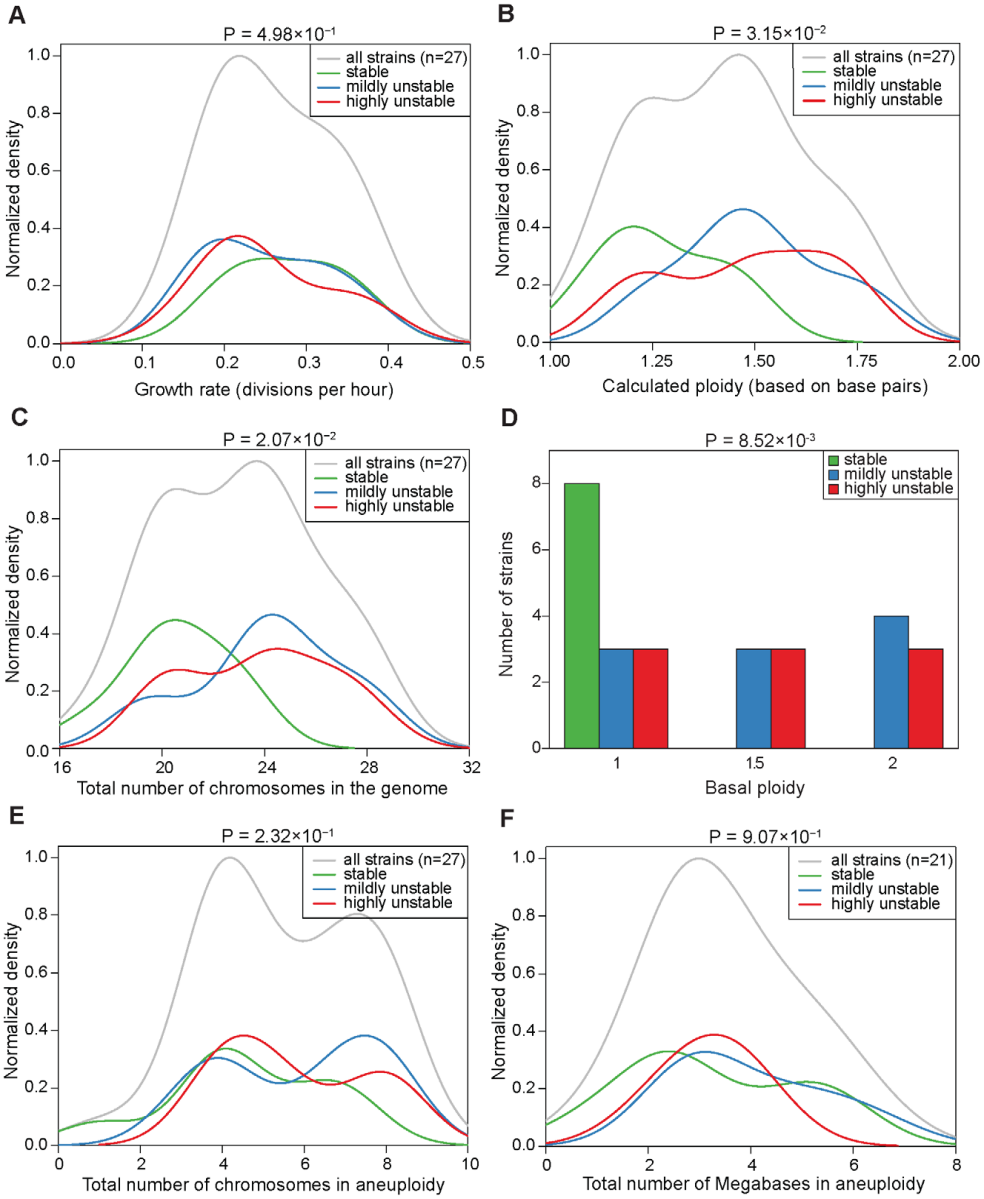
Correlations between CIN and growth rate, total genome content, or degree of aneuploidy across the aneuploid strains. (A–C and E–F) Normalized density distribution of the growth rate (A), calculated ploidy (B), total number of chromosomes in the genome (C), total number of chromosomes in aneuploidy (E), and total number of the megabases in aneuploidy (F) in the aneuploid strains in the three CIN classes. Density distribution functions were fitted on the data based on a Gaussian kernel and normalized to a maximum density of 1. The area of the three underlying curves was scaled to the relative proportion of strains in each category. (D) Distribution of the aneuploid strains based on basal ploidy. Green: S strains; blue: MU strains; red: HU strains. P-values at the top of each graph refer to the difference in means between the S and HU strains by using a Welch's t-test.

Figure 2 and Figure S7 display the resulting karyotype maps in the 27 aneuploid strains. Based on the number of independent CIN events that could be directly linked back to the original karyotypes, we qualitatively divided the 27 aneuploid strains into three classes (Figure 2A): (i) ‘stable’ (S) strains (n = 8), referring to those in which karyotype changes were not observed during our experiments (e.g. strain 245 at g30, Figure 2B); (ii) ‘mildly unstable’ (MU) strains (n = 10), in which only one chromosome copy number change event (involving either a single or multiple chromosomes) was observed at later generations (e.g. strain 226 at g30 in Figure 2C); and (iii) ‘highly unstable’ (HU) strains (n = 9) in which more than one CIN event was observed at early generations (e.g. 225 at g20 in Figure 2D). Interestingly, strains belonging to each of the three CIN categories displayed a wide range of apparent growth rates, estimated by regressing the absolute cell counts measured at the three different time points (g20, g25, g30; Figure 3A). Even though S strains exhibited a slightly higher average growth rate than HU strains, this difference was not statistically significant (P = 0.498, Figure 3A). This observation is consistent with our previous finding that stable aneuploid strains exhibit a wide range of growth abilities [15] and a recent report showing a lack of correlation between cell cycle delay and yeast artificial chromosome loss rate in yeast disomic strains [23]. Thus, it is unlikely that the observed CIN in aneuploid strains is a consequence of aneuploidy-associated fitness impairment under standard laboratory growth conditions.

### Evidence of a genome-level determinant of CIN in aneuploid strains

To ask if the aneuploidy-associated CIN might be a consequence of certain global karyotypic features, we examined the correlation between CIN and parameters such as the total number of chromosomes or base pairs in the genome, or the total number of chromosomes or base pairs in aneuploidy, etc. This analysis led to two observations. First, S strains tended to have a ploidy lower than 1.5, whereas MU or HU strains tend to have a ploidy around 1.5 or higher. Compared to HU strains, S strains showed a significantly lower base pair content (P = 3.15×10^−2^), a significantly smaller number of total chromosomes in the genome (P = 2.07×10^−2^) and a significantly lower basal ploidy (P = 8.52×10^−3^) (Figure 3B, 3C and 3D). Because the analyzed aneuploid strains had ploidy between 1N and 2N, these correlations suggest that haploid genomes with a few extra chromosomes tend to be more stable than diploids missing a few chromosomes. Second, CIN did not correlate with the number of aneuploid chromosomes: S and HU strains were significantly different neither in the total number of chromosomes in aneuploidy (P = 0.231) nor in the number of megabases in aneuploid chromosomes (P = 0.907) (Figure 3E and 3F). For this analysis, we defined basal ploidy as the integer number corresponding to the most frequently-appearing chromosome copy number in an aneuploid genome and aneuploid chromosomes as those with a copy number deviating from the basal ploidy (be it gains or losses). These observations suggest that the genome does not necessarily become more unstable as it departs further from the euploid state, however, there is a genome-level impact on CIN related to the degree of departure of the aneuploid chromosome number from the lower euploid state (in this case, the true haploid state).

### Chromosome-specific determinants of CIN

**Figure 4:**
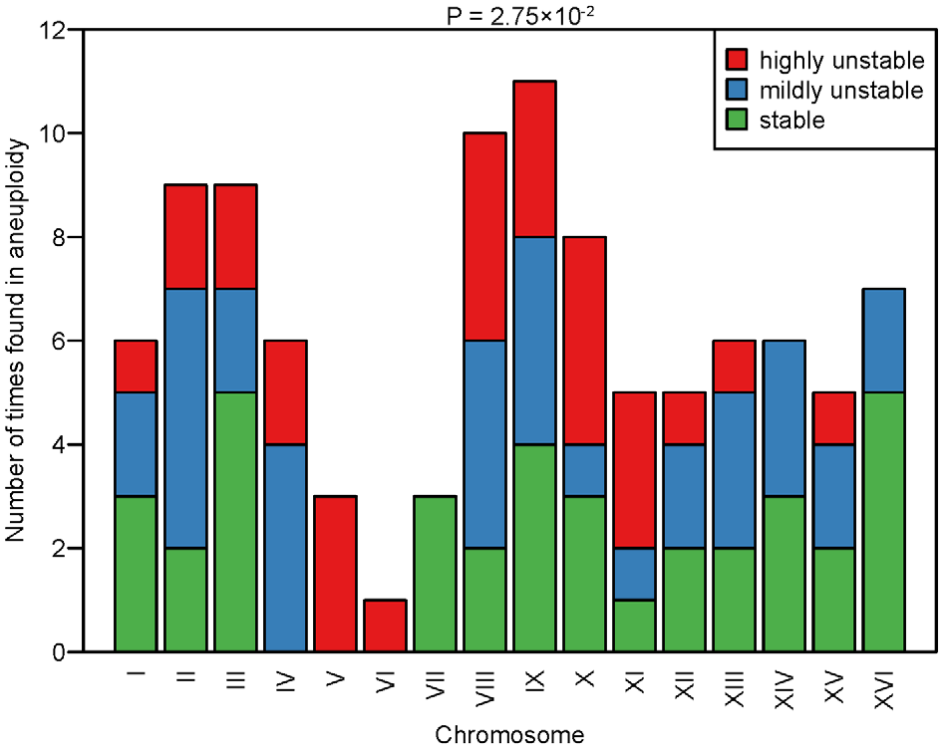
Frequency of aneuploid chromosomes in the different CIN classes. The graph shows the frequency at which each of the 16 chromosomes was found in aneuploidy (either monosomic in a diploid background or disomic in a haploid background) in the analyzed aneuploid strains. The frequency histogram is stratified across the three different CIN classes. Red: HU strains; blue: MU strains; green: S strains. P-value refers to the global association between the 16 chromosomes and the three CIN classes according to a Fisher's exact test.

We next examined the correlation between CIN and the presence of specific chromosomes in aneuploidy using the karyotyping data of the 27 strains characterized. For this analysis we focused on 21 strains for which aneuploid chromosomes could be assigned unambiguously based on basal ploidy assignment as explained above, but excluded 6 strains that had eight chromosomes with a copy number of 1 and eight chromosomes with a copy number of 2 (thus impossible to assign which chromosomes are in euploidy and which in aneuploidy). As expected, ChrVI aneuploidy was rarely found across the 21 aneuploid strains (only a single strain with ChrVI monosomy and a basal ploidy of 2N), consistent with previous reports of low tolerance of copy number imbalances of this particular chromosome, most probably due to the presence of several major cytoskeletal genes (e.g. *ACT1*, *TUB2*) on this chromosome [23], [24], [25]. We calculated the frequency at which each chromosome was present in aneuploidy across the 21 strains and searched for over- or under-representation across the three different classes of CIN (S, MU and HU) (Figure 4). In general, the frequency at which each of the 16 chromosomes was found in aneuploidy was not uniformly distributed across the three different classes of CIN (Fisher test P = 2.75×10^−2^). In particular, ChrVII aneuploidy was significantly associated with S strains (Fisher test P = 4.81×10^−2^) and ChrV aneuploidy was significantly associated with HU strains (Fisher test P = 2.03×10^−2^).

To gain molecular insights into the chromosome features associated with different levels of CIN, we analyzed enrichment of genes potentially linked to CIN on ChrV and ChrVII in comparison to other chromosomes. We used several published datasets obtained from different types of screens for chromosome instability genes, such as “genes causing increased colony sectoring when deleted” (source: *Saccharomyces* Genome Database, SGD), “genes causing increased colony sectoring when overexpressed” (SGD) or “genes causing decreased chromosome/plasmid maintenance when deleted” (SGD), as well as more comprehensive datasets such as “genes associated with chromosome instability” [26] or genes annotated with gene ontology (GO) term “chromosome segregation”. As shown in Figure S8, ChrV and ChrVII do not show exceptional or consistent over or under-representation of genes in any of these datasets.

**Figure 5:**
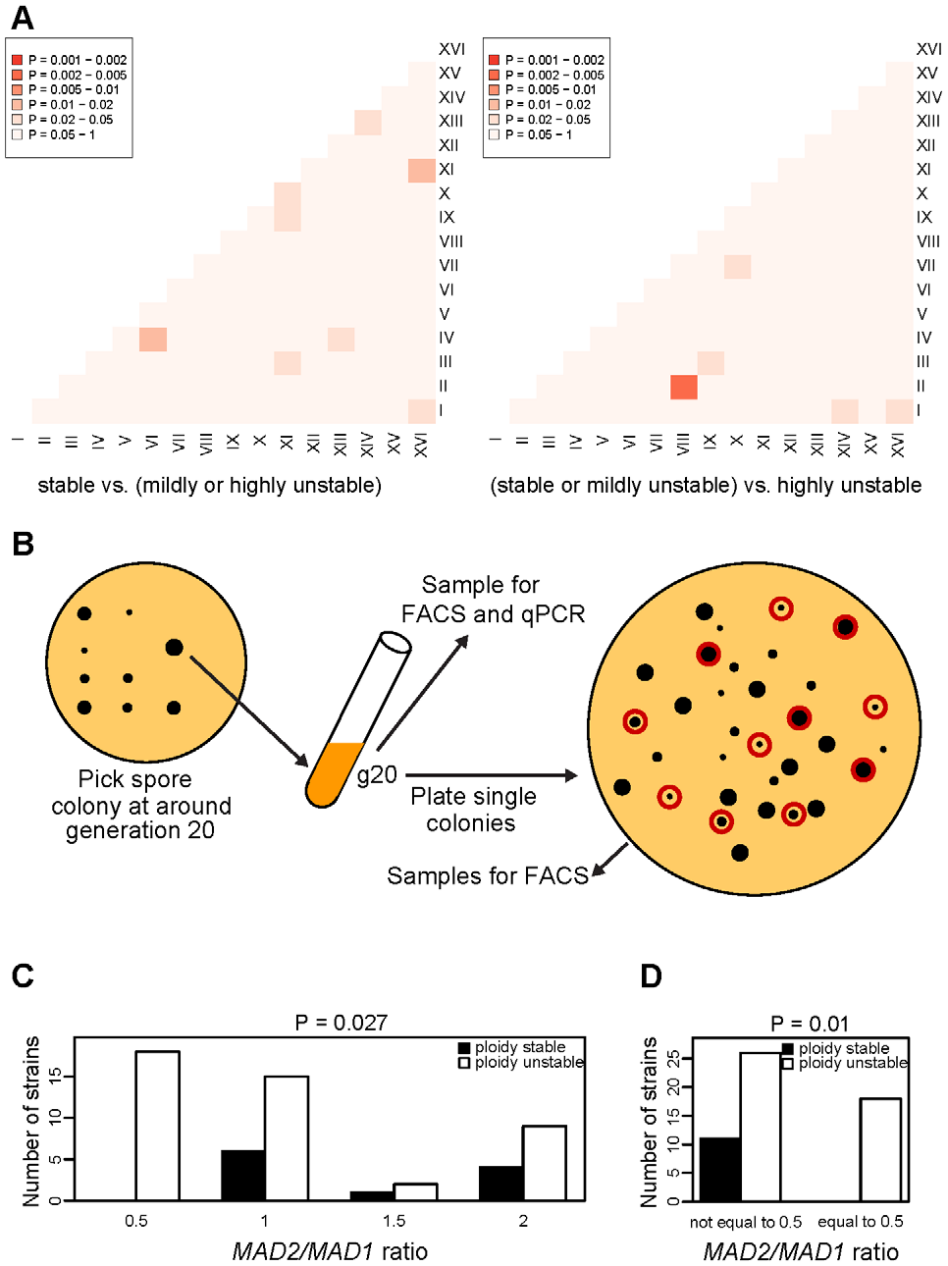
Association of chromosome copy number imbalance with CIN. (A) Enrichment of strains belonging to the combined MU and HU classes of CIN relative to the S class (left) or to the HU class of CIN relative to the combined S and MU classes (right) among aneuploid strains displaying a particular chromosome copy number imbalance, calculated for each non-redundant pair of chromosomes. The enrichment is color-coded based on p-values calculated by means of Hypergeometric tests. Darker colors indicate more significant enrichment of strains belonging to the S class of CIN (left) or HU class of CIN (right) among strains with a non-1 copy number ratio between a given pair of chromosomes. (B) Diagram illustrating the experimental design for the assessment of the relationship between CIN and *MAD2*:*MAD1* ratio in 56 freshly generated aneuploid strains from isogenic triploid sporulation. (C–D) Frequency of aneuploid strains with stable or unstable ploidy grouped by their *MAD2*:*MAD1* ratio. Ploidy-stable strains (black histograms) were identified on the basis of their low level of ploidy variation among single colonies analyzed by FACS; ploidy-unstable strains (white histograms) were identified on the basis of high ploidy variation among single colonies. *MAD2*:*MAD1* ratios were determined by qPCR and are indicated on the x-axis. P-values at the top of (C–D) graphs refer to statistical association between the stability category and the *MAD2*:*MAD1* ratio category by means of a Fisher's exact test. Aneuploid strains were divided into all four possible *MAD2*:*MAD1* ratio classes (C) or based on *MAD2*:*MAD1* ratio equal or not equal to 0.5 (D).

A lack of clear insights from the above analysis based on individual aneuploid chromosomes led us to consider the possibility that it is the relative dosage of two or multiple chromosomes rather than any particular aneuploid chromosome *per se* that affects karyotype stability. We thus performed a systematic analysis of all pair-wise combinations of the 16 chromosomes to determine if any imbalanced pairs (copy number ratio to be either 0.5 or 2, but not 1) were significantly associated with CIN. Using a hypergeometric test between two groups, relatively stable (S+MU) vs. highly unstable (HU), the imbalance between three different pairs of chromosomes were observed to distinguish the two CIN groups: ChrII vs. ChrVIII, ChrIII vs. ChrIX, and ChrVII vs. ChrX (Figure 5A, right panel). The last pair, ChrVII vs. ChrX (Hypergeometric test P<0.02), was surprising as the single chromosome analysis found ChrVII aneuploidy to be associated exclusively with karytoypically stable strains (Figure 4). A closer scrutiny found that in 2 of the 3 cases where ChrVII was in aneuploidy (strains 220 and 230, having 1 extra ChrVII with 1N basal ploidy), ChrX was also in aneuploidy with 1 extra copy, and thus their numbers were balanced. On the other hand, of the 8 aneuploid strains where ChrVII and ChrX had unequal copy numbers, 5 were highly unstable, 1 mildly unstable, and only 2 stable. These findings suggest that the effect of an individual aneuploid chromosome on CIN is dependent on the karyotypic context in which the aneuploid chromosome is present. We note that if the hypergenometric test was performed between S vs (MU+HU) groups, different pairs of chromosomes were observed whose imbalance distinguished the two groups (Figure 5A left panel). This observation suggests that there are potentially many different pairs of chromosomes whose copy number imbalance could lead to CIN.

### The effect of ChrVII and ChrX imbalance on CIN may be explained by a dosage imbalance between *MAD1* and *MAD2* genes

A recent study found that heterozygosity of *MAD2*, located on ChrX and encoding a key component of the spindle assembly checkpoint (SAC) [27], leads to partial SAC inactivation and elevated CIN in a diploid background [28]. This effect can be rescued by restoring a 1∶1 stoichiometry for the gene copy number of *MAD2* vs. *MAD1*, located on ChrVII and encoding another SAC component that physically interacts with Mad2 protein [28]. This finding implies a ratio of 0.5 for the copy numbers of *MAD2*:*MAD1* to be sufficient to induce CIN and led us to hypothesize that the association of ChrX and ChrVII imbalance with CIN may be attributed to an imbalanced *MAD2*:*MAD1* ratio of 0.5. To increase the statistical power for testing this hypothesis over the data from the 27 karyotyped strain populations, we isolated another 56 fresh aneuploid strains as the meiotic products of the same triploid strain used before. These aneuploid strains were again collected ∼20 cell divisions after tetrad dissection and an aliquot of each population was used for analysis of the relative gene copy number of *MAD1* vs. *MAD2* by qPCR on genomic DNA. Each g20 population was also plated to single colonies and after three days of growth at 23°C, 11 colonies were randomly picked from each and analyzed by FACS to determine ploidy variation (Figure 5B). This analysis found 44 of the 55 aneuploid strains to display unstable ploidies with obviously divergent ploidy profiles between the 11 colonies or Coefficient of Variation (CV) of the G1 peak positions much larger than that of the control haploid sample, whereas 11 strains were found to be ploidy-stable where the 11 picked colonies showed identical G1 peak position and CV similar to or smaller than that of the haploid control (Table S3). Genomic qPCR analysis of the g20 population samples using probes against *MAD1* and *MAD2* gene sequences found that: (i) strains with different *MAD2*:*MAD1* ratios were not uniformly distributed across the stable and unstable strains (P = 0.027, Figure 5C); and (ii) 18 aneuploid strains with a *MAD2*:*MAD1* ratio of 0.5 all fell into the ‘ploidy unstable’ category, whereas all 11 ‘ploidy stable’ aneuploid strains had a *MAD2*:*MAD1* ratio of 1 or higher, indicating a highly significant association of a *MAD2*:*MAD1* ratio of 0.5 with CIN (P = 0.01, Figure 5D). We note that 25 strains with unstable ploidy did not have the *MAD2*:*MAD1* ratio to be 0.5, indicating that a *MAD2*:*MAD1* ratio of 0.5 was sufficient but not required to cause elevated CIN. qPCR analysis using probes against the ChrVII or ChrX arm opposite to the *MAD1* or *MAD2* locus, respectively, indicated that the *MAD2:MAD1* gene copy number imbalance was indeed due to copy number imbalance between these two chromosomes (Table S3). These results demonstrate a specific case where gene dosage imbalance affecting two components of the mitotic system underlies the association between chromosome imbalance and CIN.

## Discussion

The results described above support the notion that aneuploid genomes are in general less stable than euploid genomes and prone to further karyotype changes. These findings in yeast are in agreement with recent observations that chromosomally stable pseudo-diploid human cells that accumulate aneuploid chromosomes frequently become chromosomally unstable [29]. However, our results also indicate that different aneuploid karyotypes can exhibit different degrees of CIN, with some being more stable than others, suggesting that CIN is not a necessary outcome of aneuploidy. In other words, CIN does not appear to be caused by some general property of being aneuploid *per se* but rather by determinants associated with specific aneuploid karyotypes. An advantage of our study is the carefully controlled genome variability among the strains analyzed. Because all aneuploid strains were derived from the same homozygous triploid parent and underwent minimal passage before their analysis [15], the different strains only differed in chromosome stoichiometry, minimizing the possibility that the observed CIN was due to other genetic variations or aberrations between different strains. Another advantage of our study was that CIN was assessed by examination of a wide range of spontaneously occurring karyotypic changes that include copy number gains and losses of native chromosomes. By analyzing aneuploid strains with randomly generated chromosome stoichiometries and the possibility of multiple chromosomes in aneuploid copy numbers, we were able to investigate the determinants underlying CIN in an unbiased manner and the effect of combinations of chromosomes in aneuploidy. We note, however, that our method presently does not allow analysis of those highly unstable karyotypes that quickly lead to considerable karyotype diversity within even a small population, and thus our results may not shed light on the determinants underlying extreme CIN. In addition, the qPCR-based karyotype method does not faithfully distinguish between whole-chromosome aneuploidy and partial chromosome aneuploidy and does not report on recombination events that may also be elevated in aneuploids [18].

**Figure 6:**
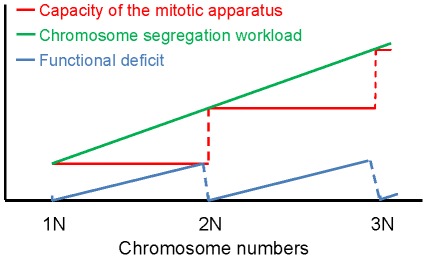
Model of discrete scaling of the functionality of the mitotic system. Schematic representation of the chromosome segregation workload (green), the capacity of the mitotic system for accurate chromosome segregation (red), and the overall functional deficit (blue) of the mitotic system (the difference between workload and mitotic capacity) as a function of increasing number of chromosome in the genome (see Discussion). The model is based on the assumption that the mitotic system increases its functionality via discrete steps only when a full set of chromosomes is gained, whereas the segregation workload increases linearly with the number of chromosomes. The resulting functional deficit explains why hypo-diploid strains are in general more chromosomally unstable than hyper-haploid strains as observed. Further studies will be required to verify whether this trend extends also to cells with a ploidy between 2N and 3N.

Consistent with a recent report [18], we did not observe any correlation between fitness and CIN among the aneuploid strains. Whereas some aneuploid strains with non-observable CIN grew relative poorly, some strains with high CIN grew relatively well. This finding suggests that CIN is not necessarily a consequence of the growth defect caused by aneuploidy under standard laboratory growth conditions, or driven by the selection for improved fitness, but may be more intrinsic to specific features of an aneuploid genome. Analysis of the correlation between CIN and different parameters associated with specific karyotypes allowed us to observe two potential determinants of CIN. On the more global level, it was surprising to find that CIN was not necessarily linked to the distance of an aneuploid karyotype from the nearest euploid state. Instead, given that the analyzed aneuploid strains had a ploidy between 1N and 2N and that each chromosome exists in a copy number of either 1 or 2, we found CIN to be significantly linked to the distance of the karyotype from the haploid state. In other words, haploids with a few extra chromosomes tend to be more stable than diploids missing a few chromosomes. As the number of aneuploid chromosomes increases from 1N towards 2N, the level of CIN tends to increase until the ploidy reaches 2N, when the level of CIN is reset to a low level (Figure 6). Future work will be necessary to test whether this trend continues beyond 2N. We propose to explain this trend by the disparity between the burden of segregating an increasing number of chromosomes and a lack of linear scaling of the capacity of the mitotic system with the aneuploid genome size. In this model, certain complex machineries, such as the kinetochore, or the mitotic spindle and the associated checkpoint mechanism, are composed of stoichiometric protein components encoded by genes distributed on all 16 chromosomes. This predicts that the functional scaling to increase the capacity of such machinery to segregate an increasing number of chromosomes from, e.g., a true haploid number might occur in a discrete rather than continuous manner and requires gaining of an entire chromosome complement (Figure 6). As such, near-diploid karyotypes are predicted to be highly unstable owing to the largest disparity between the burden of having to segregate many extra chromosomes and the capacity of the mitotic machinery that, despite the near-diploid genome size, is still working with an efficiency close to that in a haploid genome (‘functional deficit’ in Figure 6). Only upon the chromosome number reaches a true diploid state, stoichiometries are reset to their basal level and mitosis can proceed with high fidelity. We note that “scaling” in our model differs from that in a previous study on CIN in polyploid yeast cells [21]. “Scaling” in the Storchova model refers to a lack of scaling in the size of the pre-anaphase spindle with a euploid genome size (1N, 2N, 3N, 4N etc). The model intends to explain why polyploids are less chromosomally stable than haploids or diploids. Scaling in our model, on the hand, refers to the discrete increase in the functionality of the mitotic system with a linearly increasing number of chromosomes and intends to explain why certain aneuploid karyotypes are particularly unstable and why aneuploids are in general more karyotypically unstable than euploids.

Although the global trend discussed above was statistically significant, exceptions to the rule could be found when comparing instability between specific karyotypes. This suggests that karyotype-specific effects may be superimposed on the global trend. Consistent with this idea, our analysis of relative dosage between pairs of chromosomes revealed an association of CIN with dosage imbalance between specific chromosome pairs. Because the level of gene expression largely scales with gene dosage at both the transcriptome and proteome levels [15], [30], [31], chromosome copy number imbalance is likely to directly lead to altered stoichiometry of proteins that interact physically or functionally. It has been shown that an unbalanced stoichiometry in specific proteins affecting mitotic spindle function is sufficient to drive chromosome mis-segregation in cancer cell lines [20]. In yeast, one example is represented by the imbalance of *MAD1* and *MAD2* mitotic checkpoint genes [28]. Although the precise molecular explanation remains unclear, it was shown that when *MAD2* gene dosage was reduced relative to *MAD1*, such as in the case of heterozygous gene deletion, chromosome instability ensued. Stability could be restored by further deletion of a copy of *MAD1* to revert their ratio back to 1. Indeed, our data indicate that a ChrX (carrying *MAD2*) to ChrVII (carrying *MAD1*) ratio of 0.5 strongly predicts CIN. That dosage imbalance may be a prominent cause of CIN is also supported by the observation that many SAC components are deregulated at the gene expression level in several cancer cell lines without harboring sequence mutations in the corresponding genes [32]. We note that there are likely to be many gene pairs whose imbalance could lead to CIN. For example, an imbalance between ChrII and VIII is also a predictor of CIN (Figure 5A), and the chromosome passenger proteins Sli15 (INCENP) and Nbl1 are encoded on ChrII and VIII, respectively. Whereas Sli15 and Nbl1 both interact with the Aurora kinase Ipl1, Nbl1 is the yeast borealin-like and bridges the interaction between Bir1 (survivin) and Sli15 [33], [34]. It is conceivable that these chromosome passenger complex components need to be balanced in dosage to ensure proper chromosome segregation.

The flip side of the above finding is that relatively stable karyotypes may result from fortuitous but possibly complex balancing of certain key modules of the mitotic machinery. In an adaptive landscape, such metastable karyotypes may correlate with relatively stable, thus selectable, phenotypic states. This is consistent with the observation in mouse models or cancer cells that whereas moderate levels of CIN promote tumor formation or emergence of drug resistance, extremely high CIN could abate both processes [35], [36]. A recent large-scale analysis of aneuploid karyotypes in cancer cells revealed a high rate of co-occurrence of specific chromosome gains or losses [37]. While this may be explained by a requirement for balanced gene function to maintain fitness, chromosome co-gain or co-loss may also be important for achieving relatively stable cancer karyotypes in order for persistent expression of cancer phenotypes given a certain tissue microenvironment. Further, the existence of relatively stable karyotypic and phenotypic states may explain why certain chromosome aberrations in cancer are clonal [38], [39].

Finally, the observation of both global and chromosome-specific determinants of CIN may help to reconcile the chromosome/genome-centric theory vs. gene-centric theory in cancer evolution. First, our model of discrete and genome-dependent scaling of accurate chromosome segregation is consistent with the notion that complex cellular behaviors are non-linearly related to the sum of the function encoded by individual genes or even chromosomes. At the same time, the observation of different degrees of CIN associated with different aneuploid karyotypes, and more importantly with specific chromosome imbalances, highlights the exceptional impact of certain molecular components, such as Mad1 and Mad2, on the function and stability of the genome. However, even in this latter scenario, the impact of specific gene dosage is context-dependent, i.e. dependent on the dosage or activity of its partners in a manner that is potentially difficult to decode without a better knowledge of the entire cancer genome.

## Materials and Methods

### Strain generation and media

Aneuploid strains were generated as meiotic products of a homozygous triploid yeast strain as previously described [15]. All strains were cultured in either liquid or solid YEPD (Yeast Extract Peptone +2% Dextrose) media at 23°C. A list of all analyzed aneuploid strains with their karyotype information is provided in Table S1.

### Collection of population and colony samples from aneuploid cultures

Aneuploid spores were grown into colonies of ∼10^6^ cells based on preliminary experiments correlating colony size with cell number. Then the spore-derived colonies were entirely picked and resuspended into 2 mL of YEPD media. The actual cell concentration was measured using a hemocytometer and ∼200 cells were plated onto 15 cm YEPD plates. 600 µL of the culture was immediately fixed with 70% Ethanol for DNA content analysis by FACS (see below). Concomitantly a biomass corresponding to an OD_600_ = 0.3 in 300 µL was immediately frozen at −80°C for qPCR assays (see below). A part of the culture was used to prepare glycerol stocks. The remaining culture was diluted 200× with YEPD media and grown at 23°C. Cell numbers in the growing cultures were regularly monitored using the hemocytometer and ∼200 cells were spread on to YEPD plates once the cultures reached ∼25 and ∼30 cell divisions after germination. After 3–6 days incubation at 23°C, 11 colonies from each YEPD plate were randomly picked as previously described [15]. The picked colonies were inoculated into a 96-well deep-well block containing 1.5 mL YEPD media and grown overnight at 200 rpm. Each culture was harvested for FACS and qPCR analysis as described above.

### High-throughput karyotyping method

The DNA content analysis and qPCR-based karyotyping were performed essentially as previously described [15] with the only exception that FACS samples from to *MAD2*:*MAD1* ratio experiment were acquired using a MACSquant Analyzer (Miltenyi) and 6,000 events were collected for each sample. The data were analyzed using FlowJo 7.6.1. Ploidy variation analysis was performed by extracting the mode of the G1 peak position from the DNA profile of original spore and from the corresponding 11 colonies. The CV (Coefficient of Variation) was calculated between these 12 G1 peak positions and compared to the CV of 12 randomly-picked haploid colonies processed in parallel.

### *MAD2:MAD1* gene copy number ratio and apparent ploidy variation

A new set of aneuploid strains was generated as described above. When the spore colony reached ∼10^6^ cells, the colony was picked, partially harvested for FACS analysis and for *MAD2*:*MAD1* ratio determination (see below), its cell number was determined at the hemocytometer and ∼200 cells were plated into fresh YEPD plates. Once colonies became visible, 11 randomly selected colonies were picked and processed for FACS analysis as described above. When aligning the DNA profile of the original spore to the DNA profiles of the corresponding 11 colonies, the following criteria were applied: (i) those strains with obvious heterogeneous and noisy DNA profiles were classified as unstable; (ii) strains with clean DNA profiles and similar ploidy were subjected to ploidy variation analysis as described above. A strain was classified as ‘ploidy stable’ only if it showed similar or smaller CV compared to that of a haploid control strain processed in parallel. Samples harvested for *MAD2:MAD1* copy number ratio determination were resuspended in 20 µL PBS (pH = 7.4) containing 50 µg/µl Zymolyase 100T (US Biological) and incubated at 37°C for 30 min. These samples were diluted 1∶200. To prepare qPCR reactions, 2 µl of these dilutions were combined with 8 µl 1× Perfecta SYBR Green Mix (Quanta) at 500 nM for forward and reverse primers in technical triplicates on a CAS-4200 robot (Corbett) and run on an ABI 7900HT cycler with the following cycling conditions: 95°C for 5 min, then 40 cycles of 95°C for 15 s followed by 60°C for 1 min. Ct values were obtained using SDS 2.4 software (ABI). The ratio of *MAD2:MAD1* copy number presented in Figure 5C and 5D were calculated using the NRQ method in qbasePLUS version 2.0 software (Biogazelle) by setting either *MAD1* or ChrVII as the endogenous control and scaling all samples to wild type haploid. All primers used in this study are listed in Table S2.

### Statistical analysis and computer simulations

All statistical analyses and computer simulations were performed in the R environment for statistical computing. Difference in means was evaluated by means of two-sided unpaired Welch's t-test, association between categorical data by Fisher's Exact Test for count data, overlap between subsets by Hypergeometric test and difference between empirical cumulative distribution functions by Kolmogorov-Smirnov test. Results were considered significant if P<0.05.

Simulation of the expected fraction of cells with deviant karyotype as a function of generation time and chromosome mis-segregation rate was performed as follows. A seeding cell was represented as a vector of length 16, each element of which represented the copy number of one of the 16 yeast chromosomes. At each generation, each cell was allowed to self-duplicate and during this process each of the 16 duplicated chromosomes was allowed to mis-segregate with a given probability, resulting in one daughter inheriting both copies and the other daughter not inheriting any copy of that particular chromosome. At the end of each generation, cells that lost all copies of any given chromosome were discarded as dead cells. Every time the simulated colony reached >100,000 cells, a random sampling of 100,000 cells was used to simulate the next generation to limit computational complexity. As a control, the same simulation was performed using different cell number cutoffs without significant differences (data not shown).

Simulation of the expected distribution of apparent ploidy of spores from triploid meiosis was performed as follows. As above, random spores were represented as vectors of length 16, in which each element represented one of the 16 yeast chromosomes and having identical and independent probability of being of copy number ‘1’ or ‘2’. The apparent ploidy of each random spore was calculated based on the known length in base pairs of each of the 16 yeast chromosomes. 10,000 independent simulations were performed, in each of which 41 random spores were generated, i.e. the same number as the experimentally determined ones.

### Karyotype network reconstruction and determination of level of CIN

Chromosome copy number data from both the g20 population and from the 11 colonies analyzed at either g20, g25 or g30 were combined into a matrix of size 12×16, in which each row represented one of the 12 ‘individuals’ and each column represented one of the 16 ‘loci’ carrying one of two ‘alleles’, corresponding to the two copy number states (i.e. ‘1’ and ‘2’). This matrix was used as input for the Network software (version 4.5.1.6.), which reconstructed the most likely karyotype network by minimizing the number of allelic changes (here: chromosome copy number gain/loss events) across the entire map. Karyotype changes involving more than one chromosome copy number change were scored conservatively as a single event, as we ignored whether the multiple chromosome mis-segregations occurred in a single erroneous mitosis or multiple subsequent mitotic events. Also, karyotype changes unlikely to have originated directly from the inferred original spore karyotype, but more likely to have originated from one of its karyotypically deviant progeny according to the reconstructed karyotype network, were not counted for the determination of the level of CIN of the ancestral karyotype, as they would be more reflective of the level of CIN of one of its karyotypic deviants as opposed to the level of CIN of the ancestral karyotype.

## Supporting Information

Figure S1 Diagram explaining the number of aneuploid strains analyzed at each step of this study. Reasons for discarding specific strains for subsequent analyses are given on the right.
(PDF)

Figure S2 Computer simulations of fraction of cells with deviant karyotype as a function of chromosome mis-segregation rates. (A–B) Fraction of cells with deviant karyotypes after 20 (A) or 30 (B) generations. Chromosome mis-segregation rates are indicated on the x-axis whereas the percentage of cells with deviant karyotype is indicated on the y-axis. Box-plots represent median (thick horizontal bar), inter-quartile range (rectangular square) and outliers (circles) of 30 independent simulations. See Materials and Methods for details on the computer simulation.
(PDF)

Figure S3 DNA content profiles of all 47 g20 populations analyzed by FACS. FACS profiles of the indicated strains from the g20 population samples are shown in separate panels. The profile of a haploid control strain run in parallel is superimposed on each plot.
(PDF)

Figure S4 Comparison of observed and expected distribution of apparent ploidies from aneuploid spores obtained by triploid meiosis. Apparent ploidy data was derived from the mode of the G1 peak position (measured by FACS analysis) of all 47 analyzed viable spores obtained by meiosis of a homozygous triploid strain in comparison to the mode of the G1 peak position of a control haploid strain run in parallel. Simulated ploidy data was obtained by computer-generated random karyotypes as explained in the Materials and Methods. Empirical cumulative distribution functions are shown for both datasets and their statistical difference was tested by means of a Kolmogorov-Smirnov test.
(PDF)

Figure S5 Karyotypes of the 41 g20 populations. Each panel shows the absolute chromosome copy numbers determined for each of the 41 karyotyped strains, by combining information from high-throughput FACS and qPCR assays. The 27 strains further processed for analysis, the 6 strains discarded because of redundancy (i.e. part of a pair of siblings with identical karyotype) and the 8 strains discarded because of excessive heterogeneity are each labeled accordingly.
(PDF)

Figure S6 FACS profiles of the g20 population sample and 11 g20 colony samples. FACS profiles are overlaid and the coefficient of variation (CV) is calculated between the G1 peaks of the 12 samples. (A) Wild type haploid G1 peaks and CV; (B) an example of a strain (s203) with sharp G1 peaks and low CV; (C) an example of a strain (s236) with wide G1 peaks and large CV.
(PDF)

Figure S7 Karyotype information and karyotype networks of all 27 analyzed aneuploid strains. For all 27 analyzed aneuploid strains, karyotype makeups and reconstructed karyotype networks are shown. The number of CIN events used to qualitatively classify the aneuploid strains is shown on the right. See legend of Figure 2 for details on data presentation. Note that there exist two equally probable karyotype networks for strain 252, however in both cases the number of CIN events directly linked back to the original karyotype are the same hence its CIN classification is not affected. Alternative network is indicated by dashed lines.
(PDF)

Figure S8 Distribution of genes implicated in CIN across the 16 yeast chromosomes. For each of the 16 yeast chromosomes, the y coordinate represents the number of genes belonging to a specific class (identified on the y-axis of the diagram) present on the chromosomes and the x coordinate represents the total number of protein-coding genes on the same chromosome. The dashed line represents the expected number of genes in each class based on the assumption of uniform distribution across the 16 chromosomes. Chromosome V and chromosome VII are highlighted in red and green respectively.
(PDF)

Table S1 Aneuploid strains used in this study and associated chromosome copy numbers determined by FACS and qPCR.
(XLS)

Table S2 Primers used for qPCR determination of *MAD1*:*MAD2* ratio from genomic DNA of 56 freshly generated aneuploid strains.
(XLS)

Table S3 *MAD1*:*MAD2* ratio and ploidy stability of 56 freshly generated aneuploid strains.
(XLS)
