# Supplementary figures and images for: Karyotypic Determinants of Chromosome Instability in Aneuploid Budding Yeast
Source: PLoS Genet. 2012 May 17;8(5):e1002719. doi: 10.1371/journal.pgen.1002719 (PMC3355078; doi:10.1371/journal.pgen.1002719)

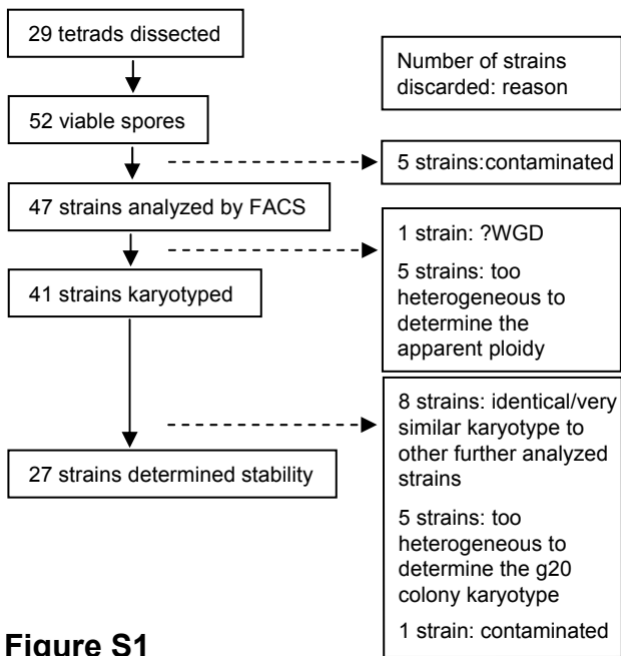

Supplement: Figure S1 — Diagram explaining the number of aneuploid strains analyzed at each step of this study. Reasons for discarding specific strains for subsequent analyses are given on the right. (PDF) [file pgen.1002719.s001.pdf]

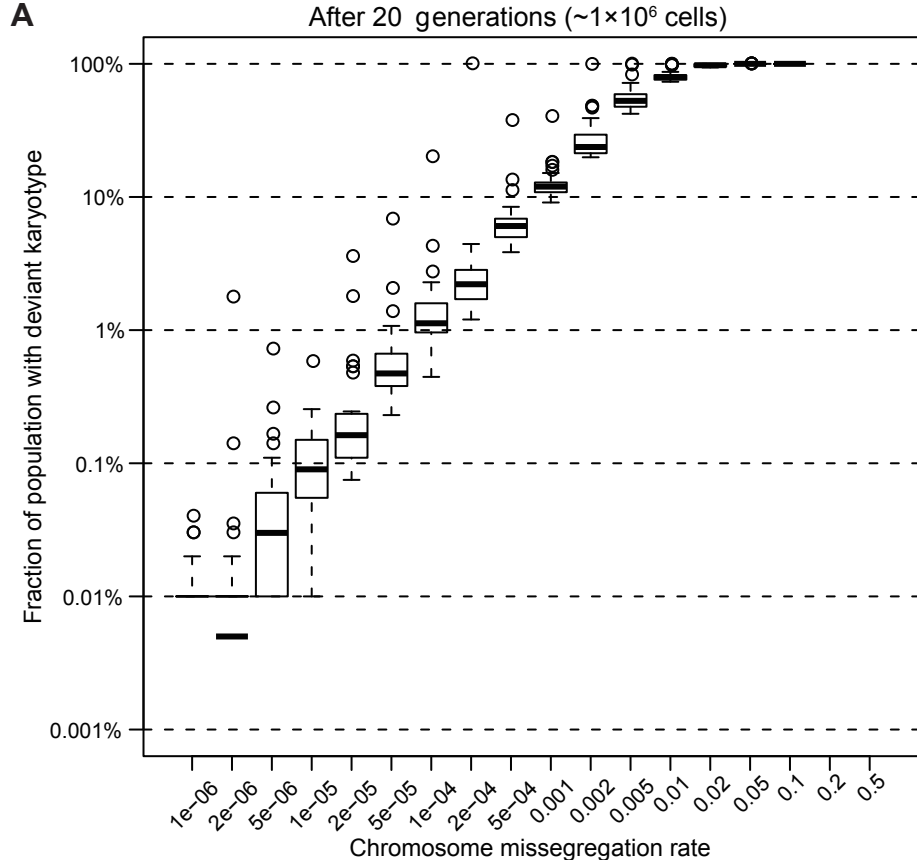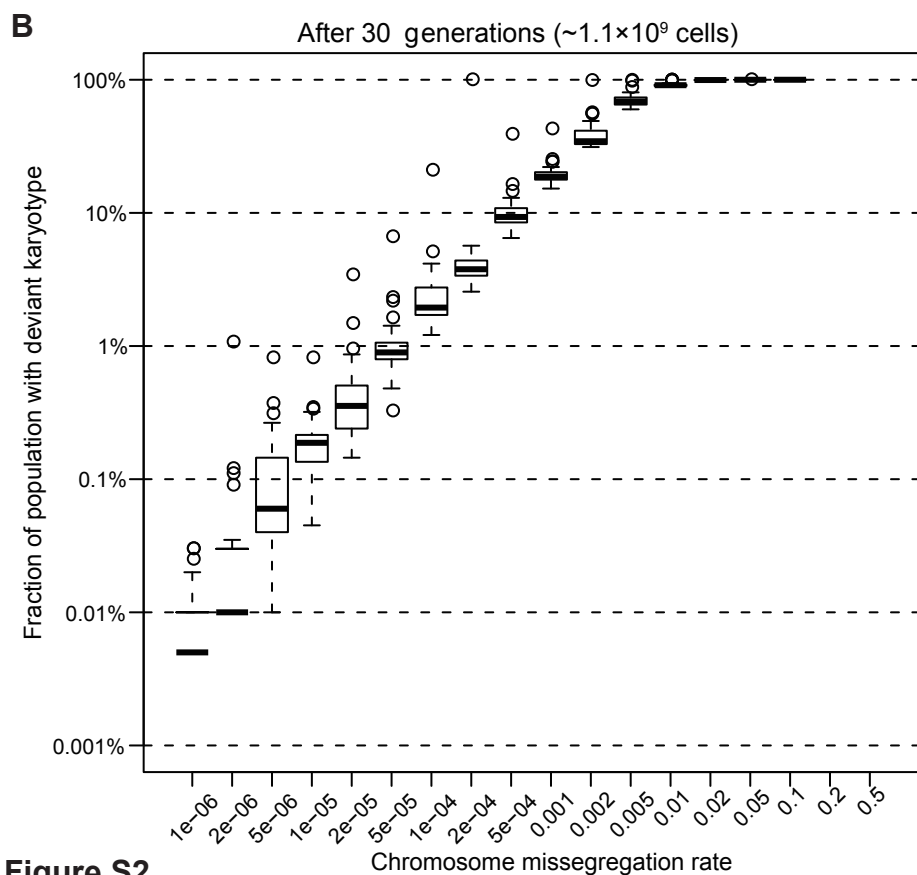

**Figure S2**

Supplement: Figure S2 — Computer simulations of fraction of cells with deviant karyotype as a function of chromosome mis-segregation rates. (A–B) Fraction of cells with deviant karyotypes after 20 (A) or 30 (B) generations. Chromosome mis-segregation rates are indicated on the x-axis whereas the percentage of cells with deviant karyotype is indicated on the y-axis. Box-plots represent median (thick horizontal bar), inter-quartile range (rectangular square) and outliers (circles) of 30 independent simulations. See Materials and Methods for details on the computer simulation. (PDF) [file pgen.1002719.s002.pdf]

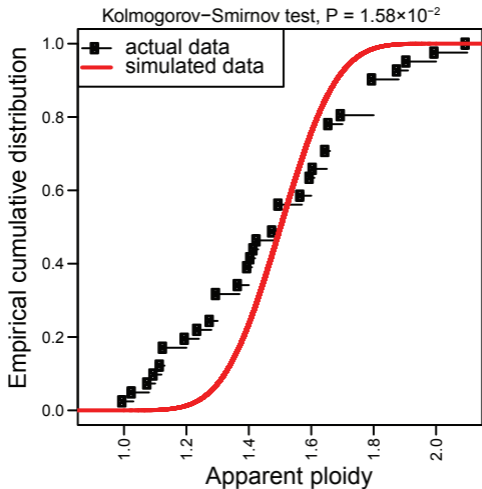

**Figure S4**

Supplement: Figure S4 — Comparison of observed and expected distribution of apparent ploidies from aneuploid spores obtained by triploid meiosis. Apparent ploidy data was derived from the mode of the G1 peak position (measured by FACS analysis) of all 47 analyzed viable spores obtained by meiosis of a homozygous triploid strain in comparison to the mode of the G1 peak position of a control haploid strain run in parallel. Simulated ploidy data was obtained by computer-generated random karyotypes as explained in the Materials and Methods. Empirical cumulative distribution functions are shown for both datasets and their statistical difference was tested by means of a Kolmogorov-Smirnov test. (PDF) [file pgen.1002719.s004.pdf]

**A** haploid G1 peak CV=2.30%

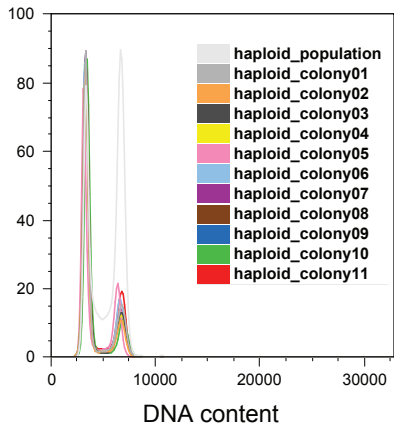

**B** Strain 203 G1 peak CV=3.67%

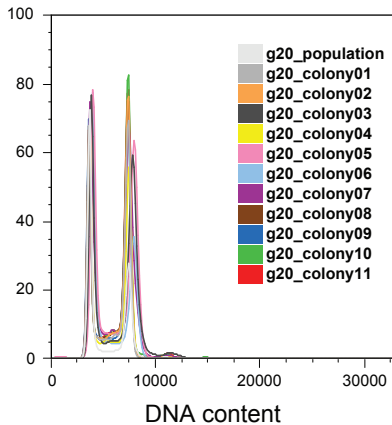

**C** Strain 236 G1 peak CV=29.27%

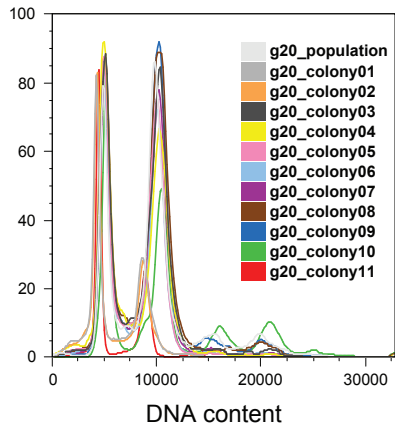

**Figure S6**

Supplement: Figure S6 — FACS profiles of the g20 population sample and 11 g20 colony samples. FACS profiles are overlaid and the coefficient of variation (CV) is calculated between the G1 peaks of the 12 samples. (A) Wild type haploid G1 peaks and CV; (B) an example of a strain (s203) with sharp G1 peaks and low CV; (C) an example of a strain (s236) with wide G1 peaks and large CV. (PDF) [file pgen.1002719.s006.pdf]

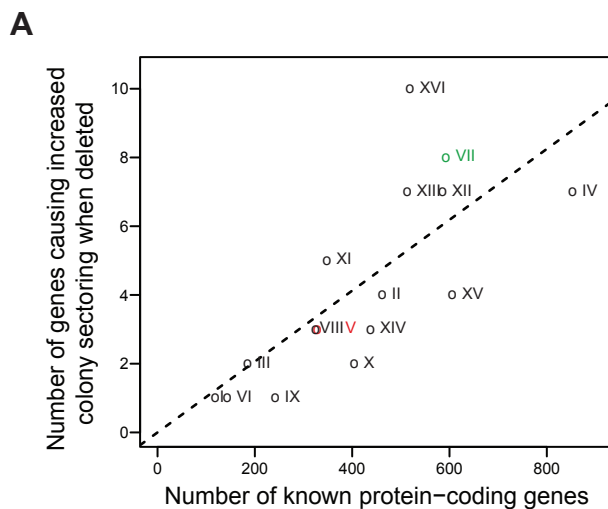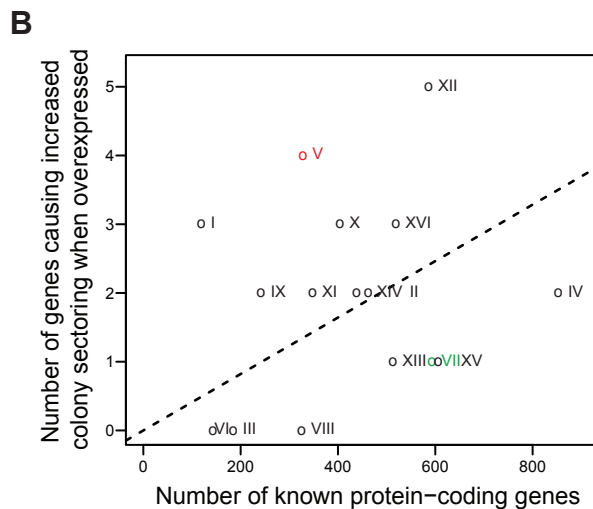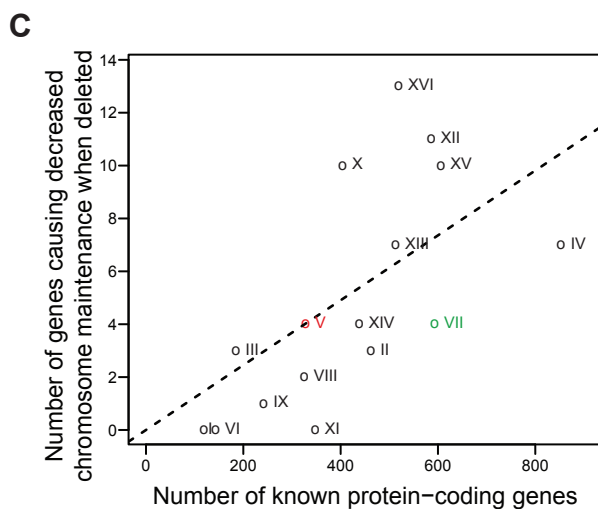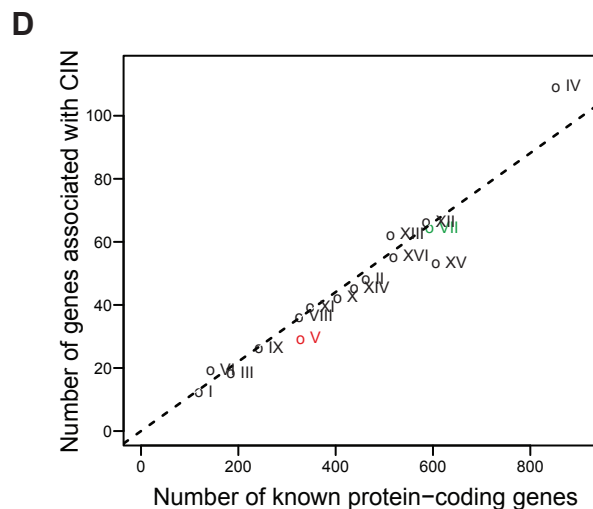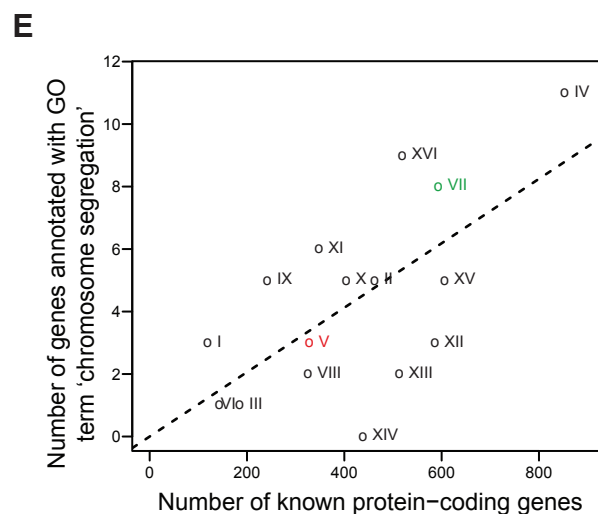

**Figure S8**

Supplement: Figure S8 — Distribution of genes implicated in CIN across the 16 yeast chromosomes. For each of the 16 yeast chromosomes, the y coordinate represents the number of genes belonging to a specific class (identified on the y-axis of the diagram) present on the chromosomes and the x coordinate represents the total number of protein-coding genes on the same chromosome. The dashed line represents the expected number of genes in each class based on the assumption of uniform distribution across the 16 chromosomes. Chromosome V and chromosome VII are highlighted in red and green respectively. (PDF) [file pgen.1002719.s008.pdf]
