# Supplementary material for: Karyotypic Determinants of Chromosome Instability in Aneuploid Budding Yeast
Source: PLoS Genet. 2012 May 17;8(5):e1002719. doi: 10.1371/journal.pgen.1002719 (PMC3355078; doi:10.1371/journal.pgen.1002719)
Supplement: Figure S3 — DNA content profiles of all 47 g20 populations analyzed by FACS. FACS profiles of the indicated strains from the g20 population samples are shown in separate panels. The profile of a haploid control strain run in parallel is superimposed on each plot. (PDF) [file pgen.1002719.s003.pdf]

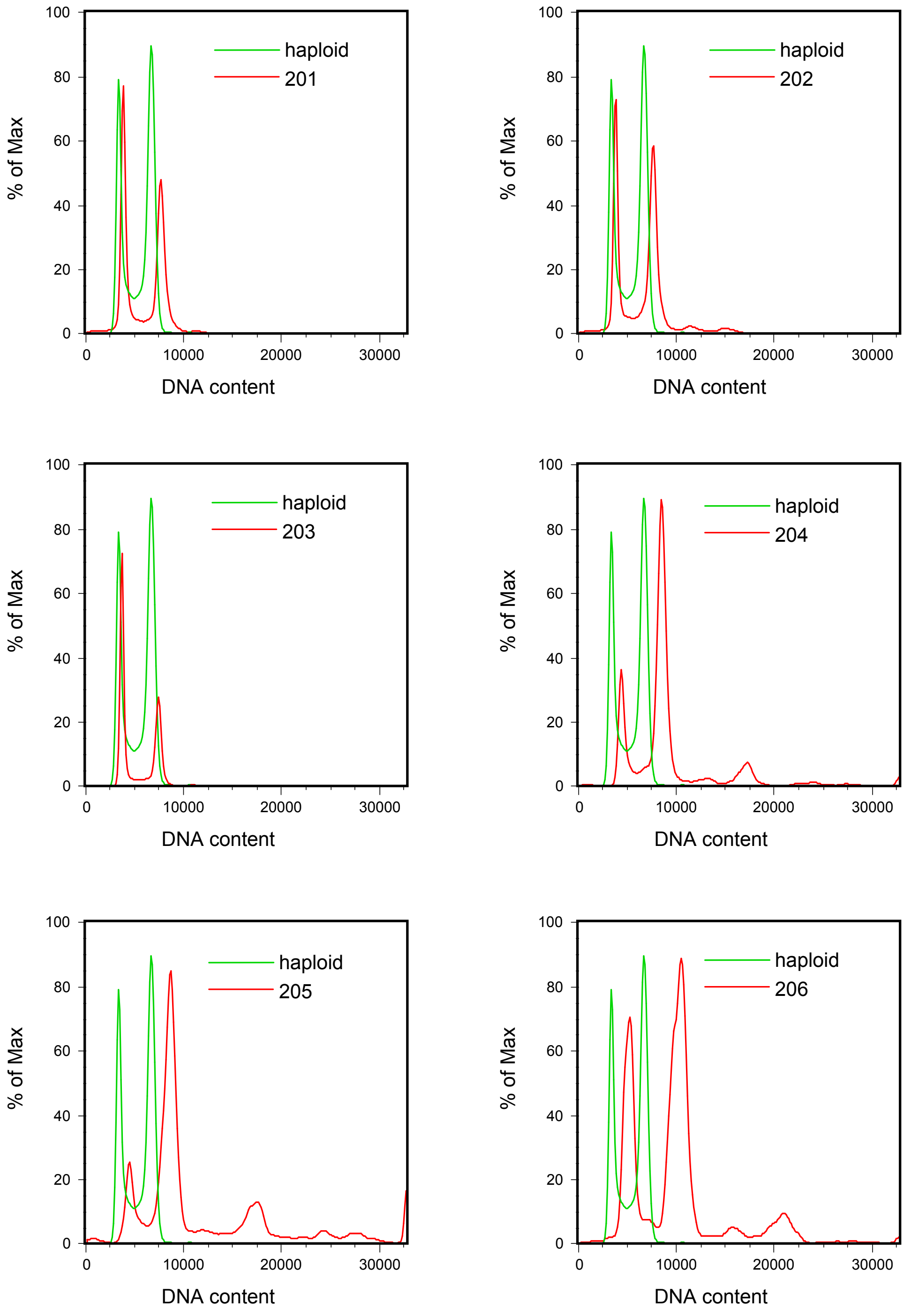

Figure S3 (page 1)

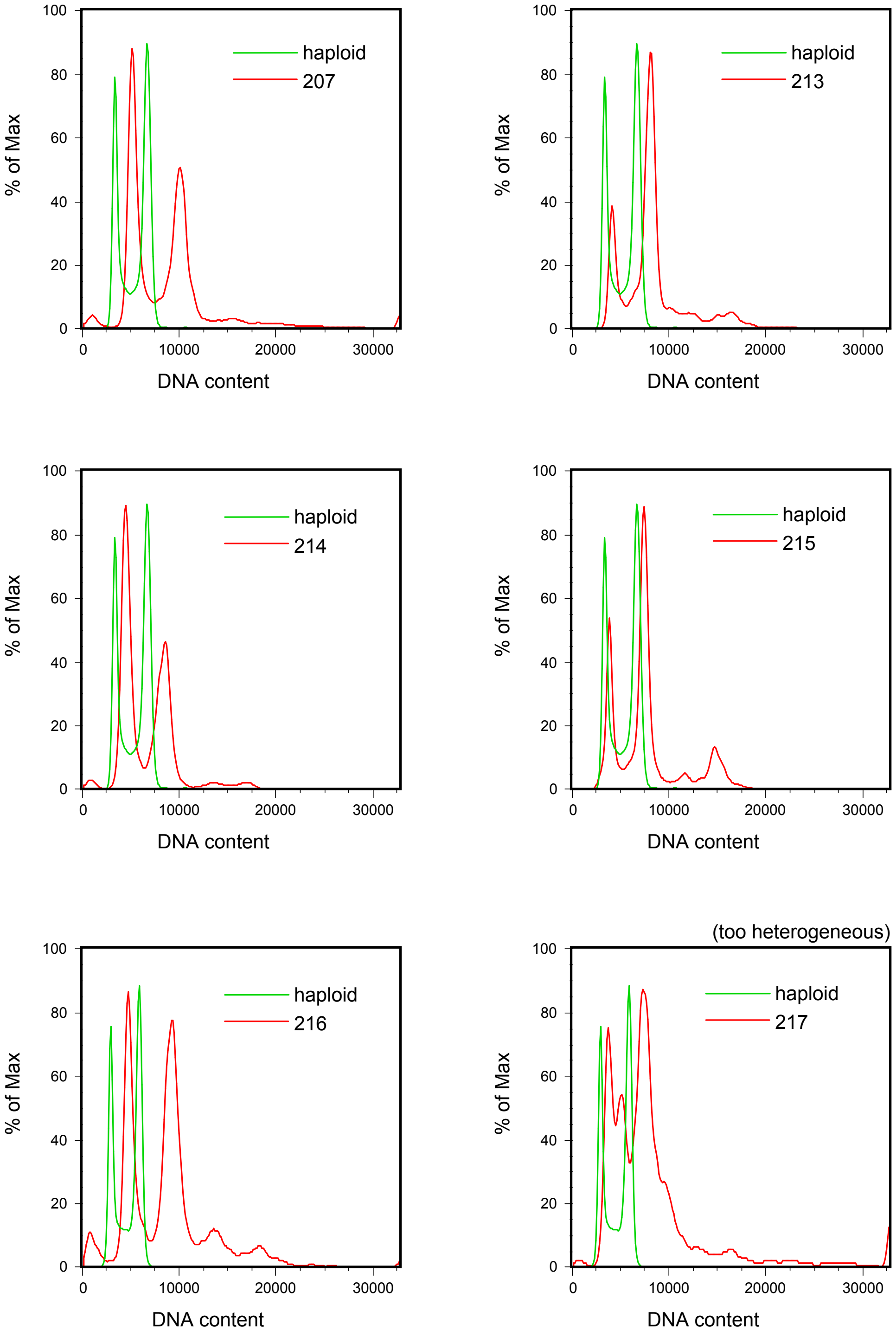

Figure S3 (page 2)

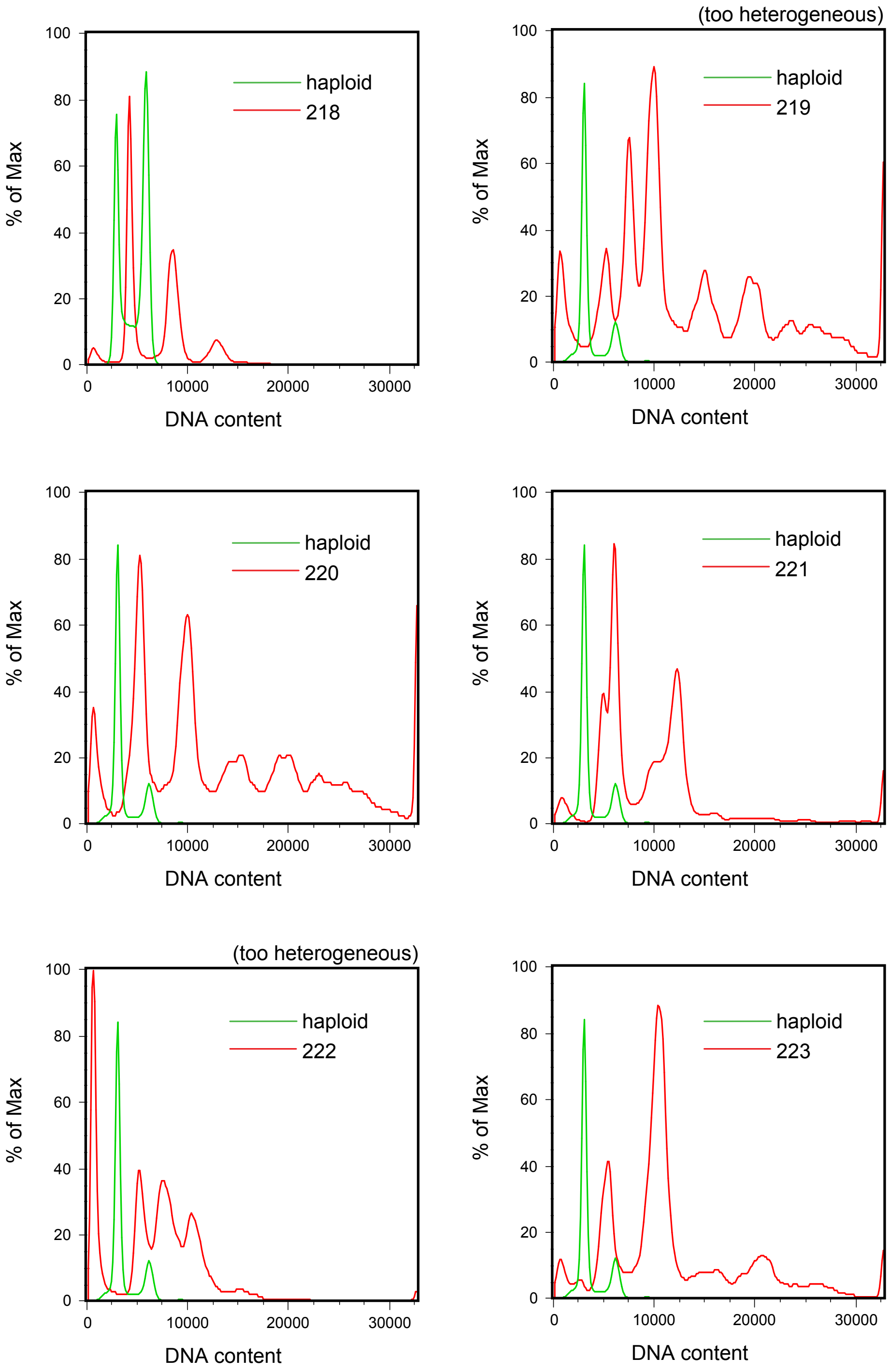

Figure S3 (page 3)

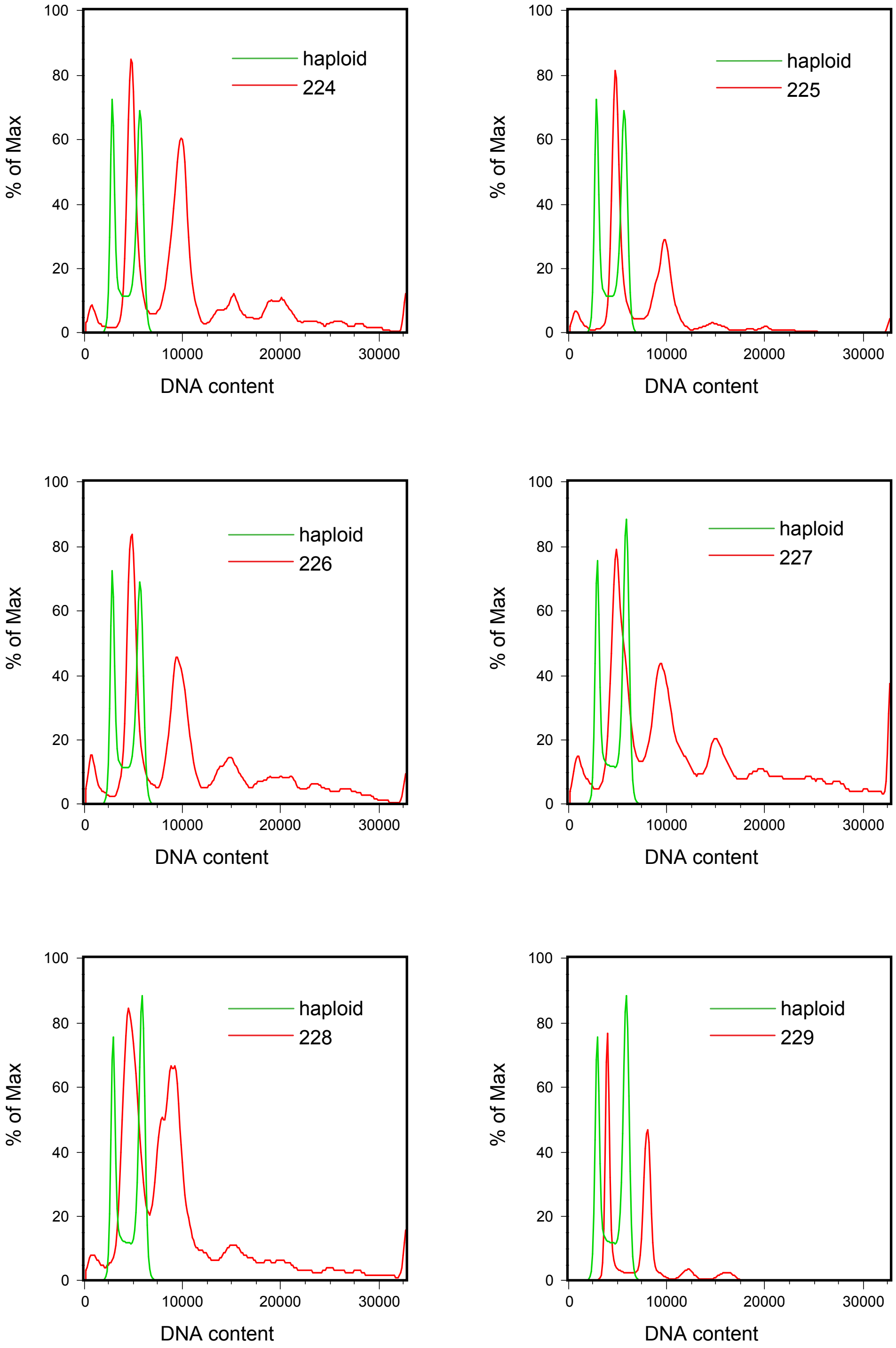

Figure S3 (page 4)

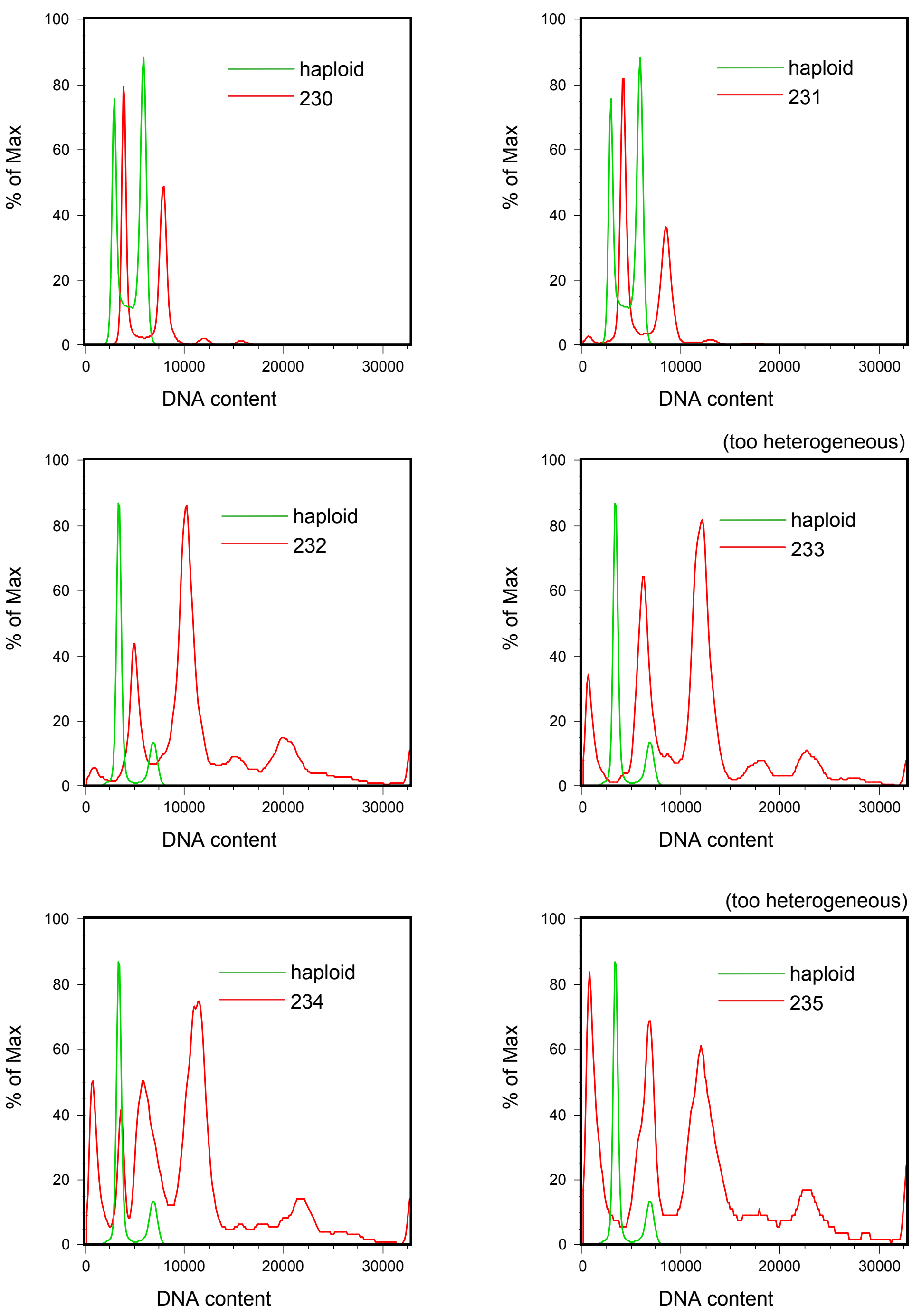

Figure S3 (page 5)

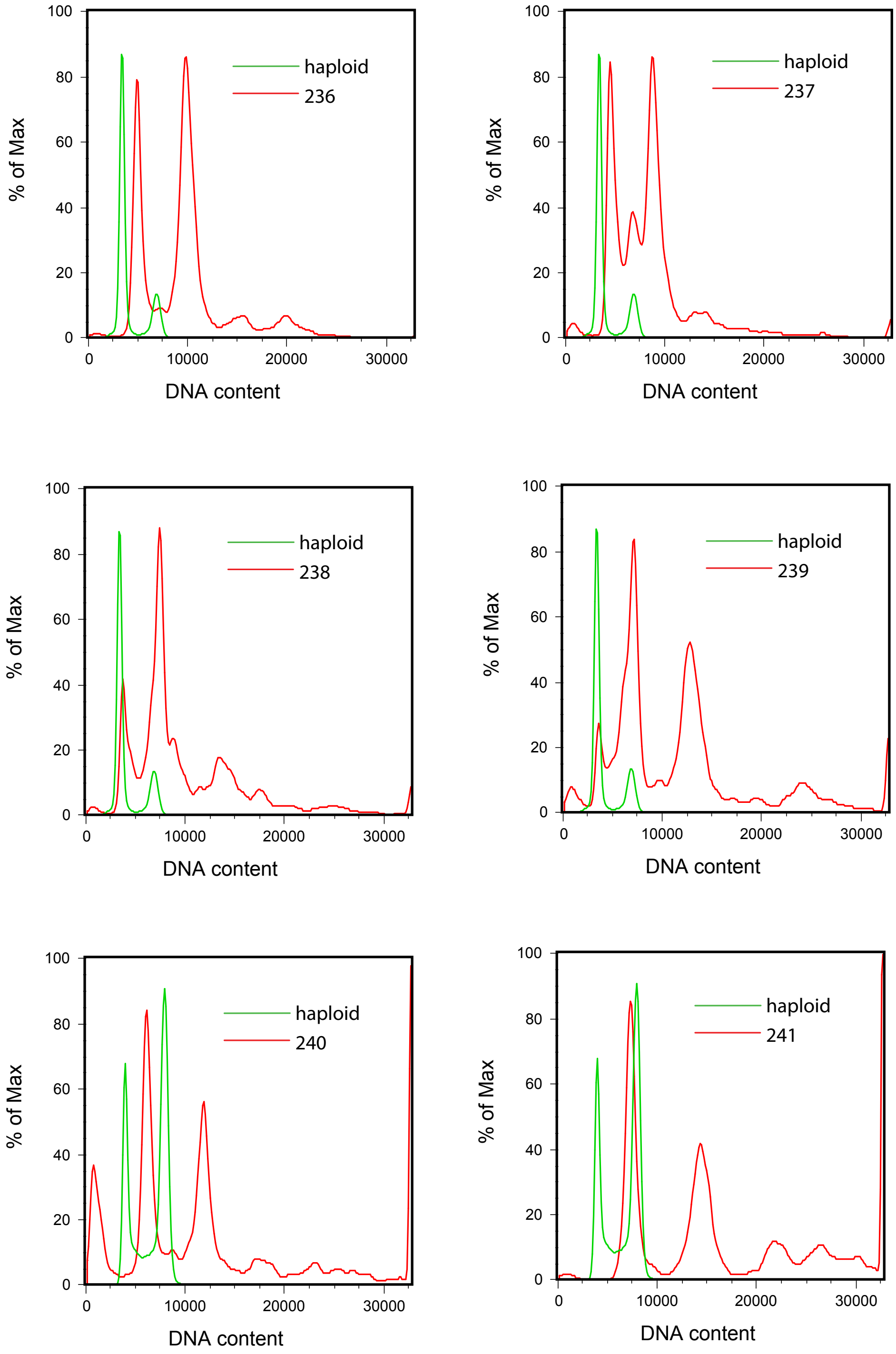

Figure S3 (page 6)

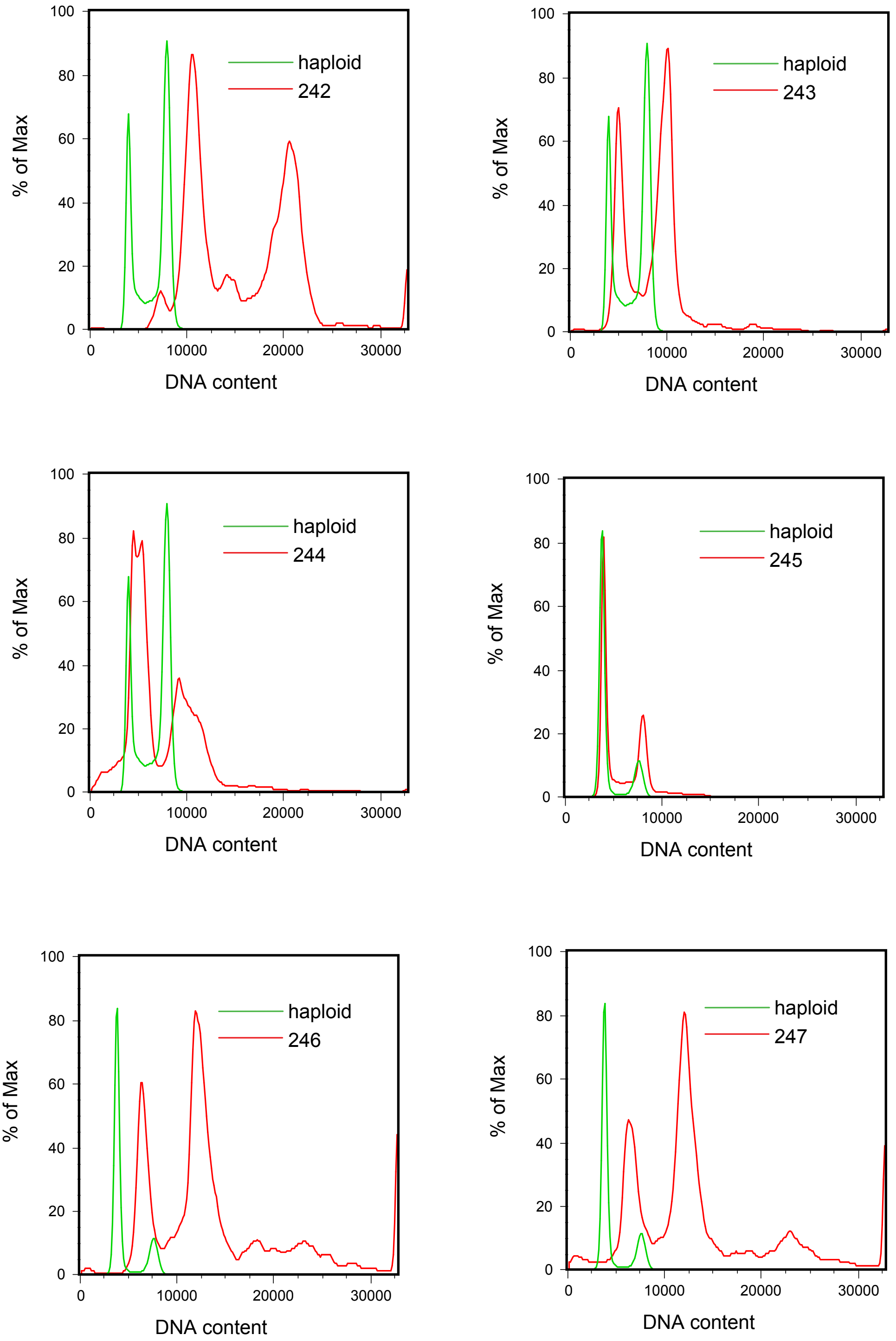

Figure S3 (page 7)

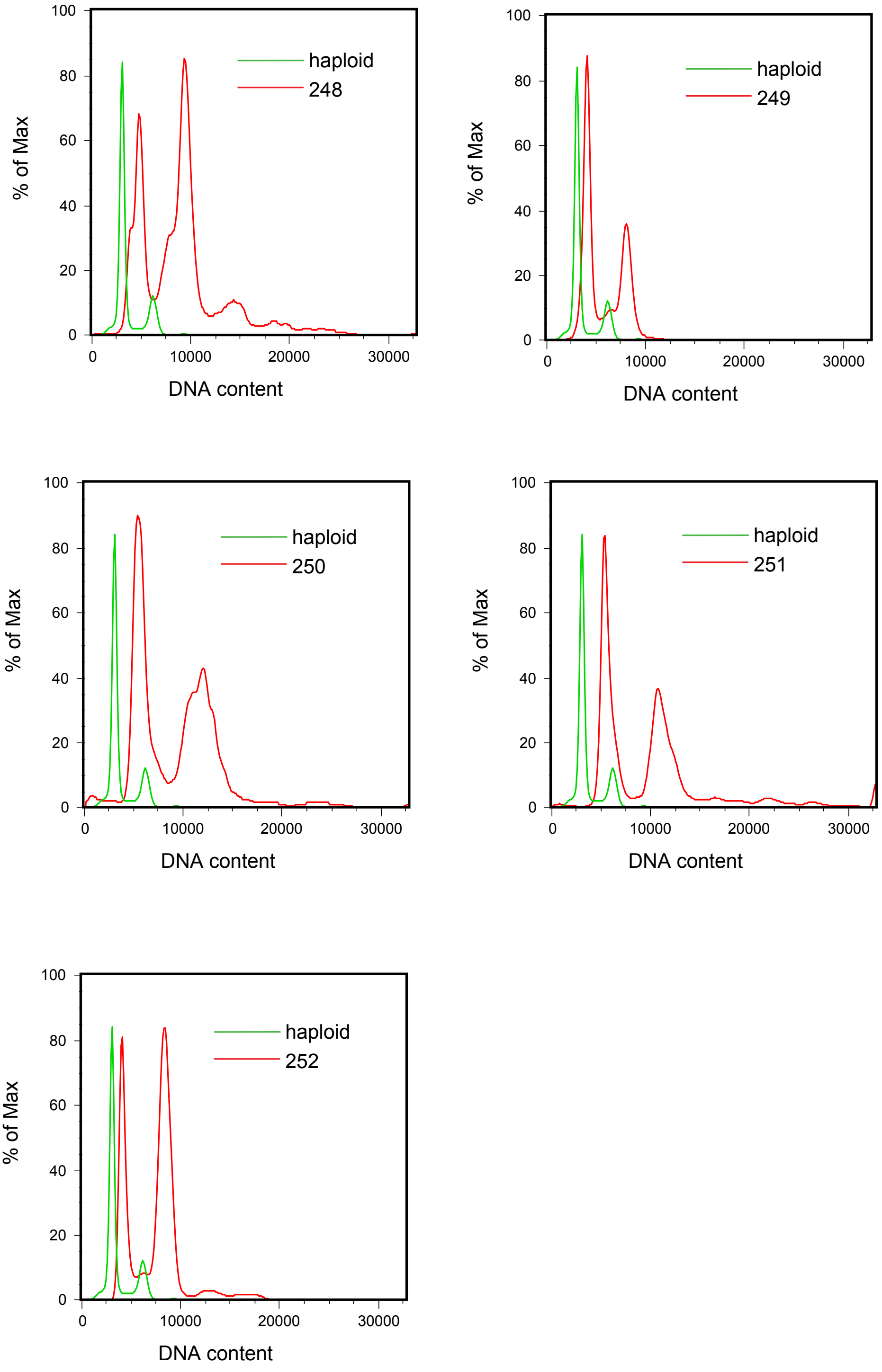

Figure S3 (page 8)
