# Supplementary material for: Karyotypic Determinants of Chromosome Instability in Aneuploid Budding Yeast
Source: PLoS Genet. 2012 May 17;8(5):e1002719. doi: 10.1371/journal.pgen.1002719 (PMC3355078; doi:10.1371/journal.pgen.1002719)
Supplement: Figure S5 — Karyotypes of the 41 g20 populations. Each panel shows the absolute chromosome copy numbers determined for each of the 41 karyotyped strains, by combining information from high-throughput FACS and qPCR assays. The 27 strains further processed for analysis, the 6 strains discarded because of redundancy (i.e. part of a pair of siblings with identical karyotype) and the 8 strains discarded because of excessive heterogeneity are each labeled accordingly. (PDF) [file pgen.1002719.s005.pdf]

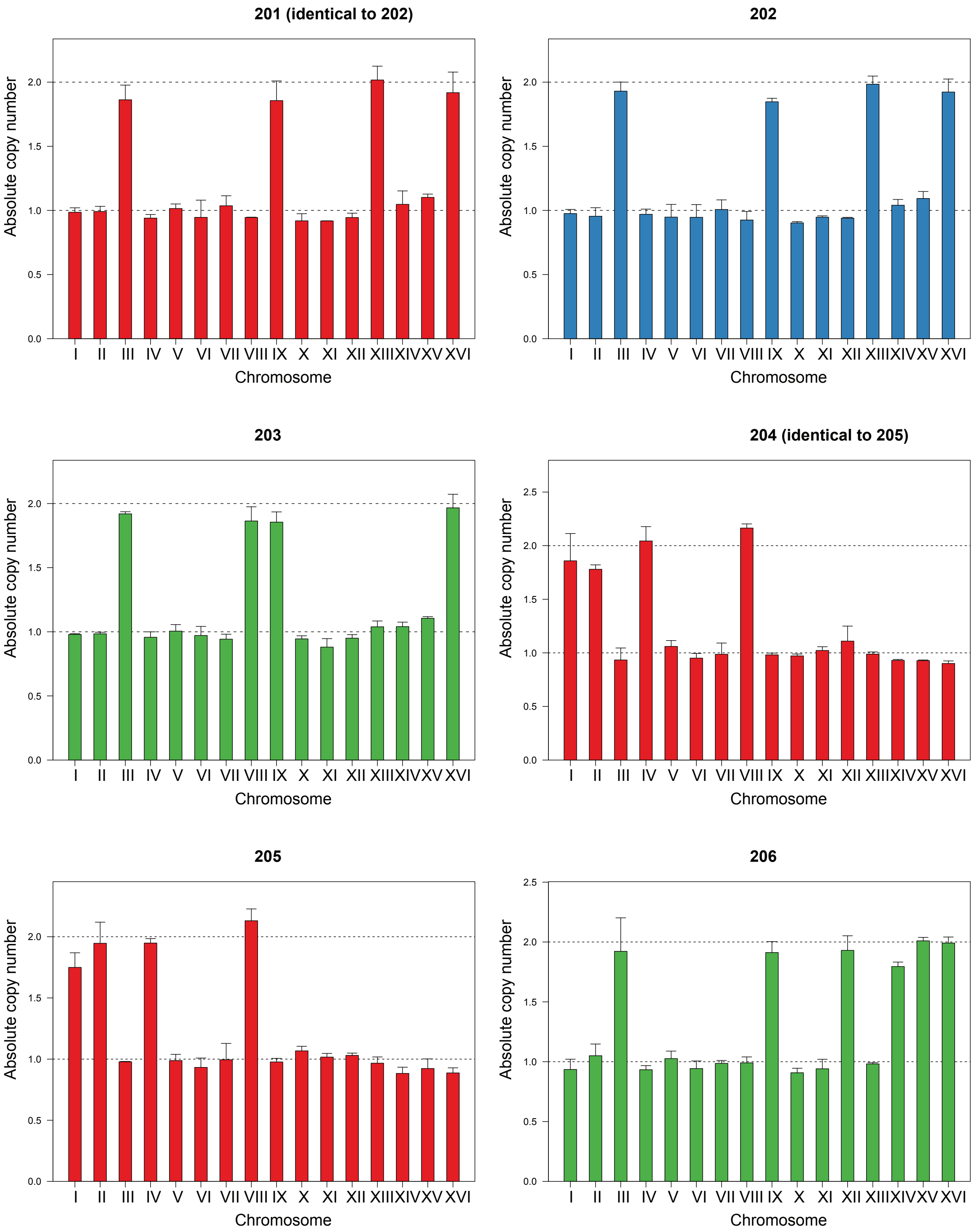

Figure S5 (page 1)

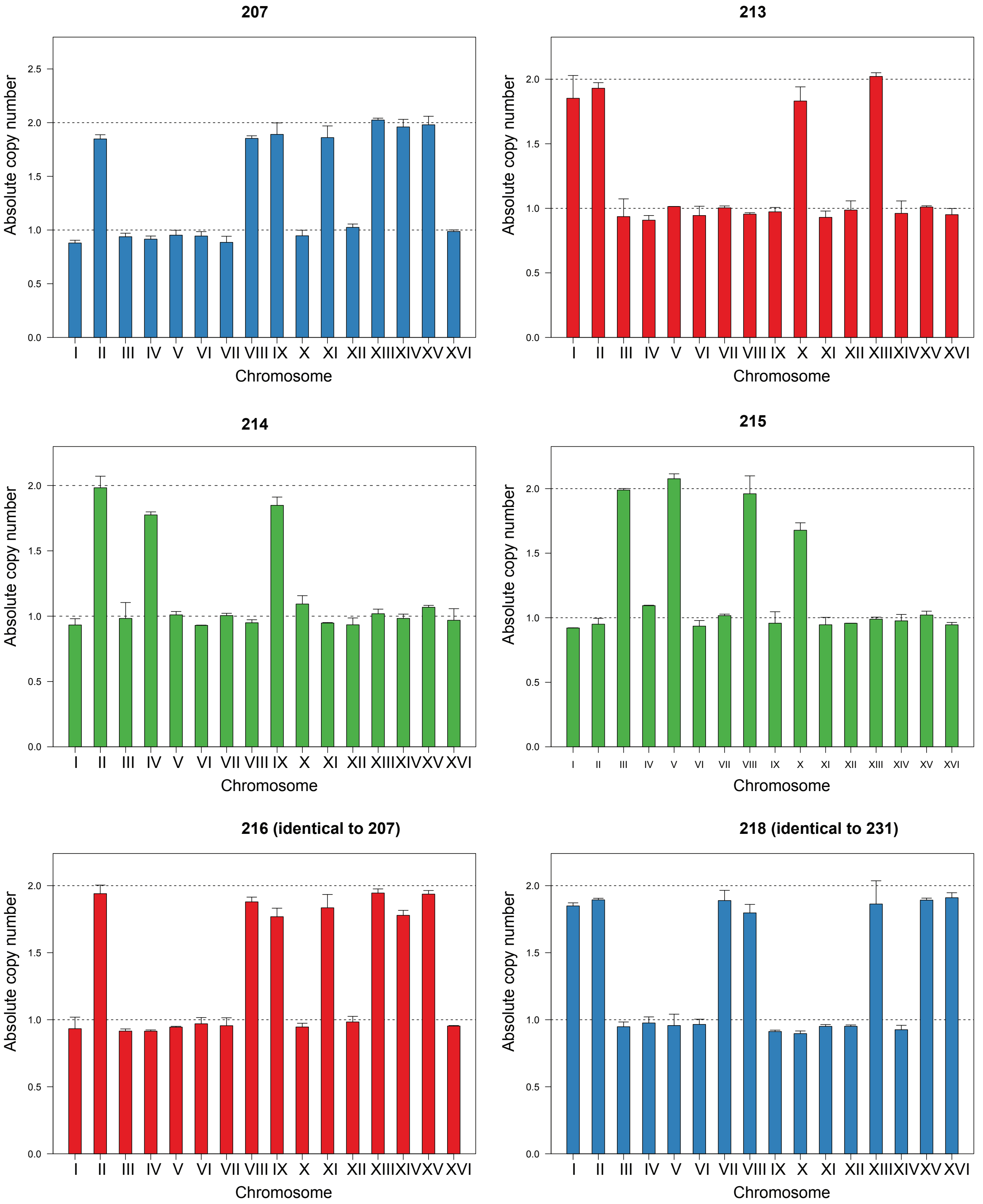

Figure S5 (page 2)

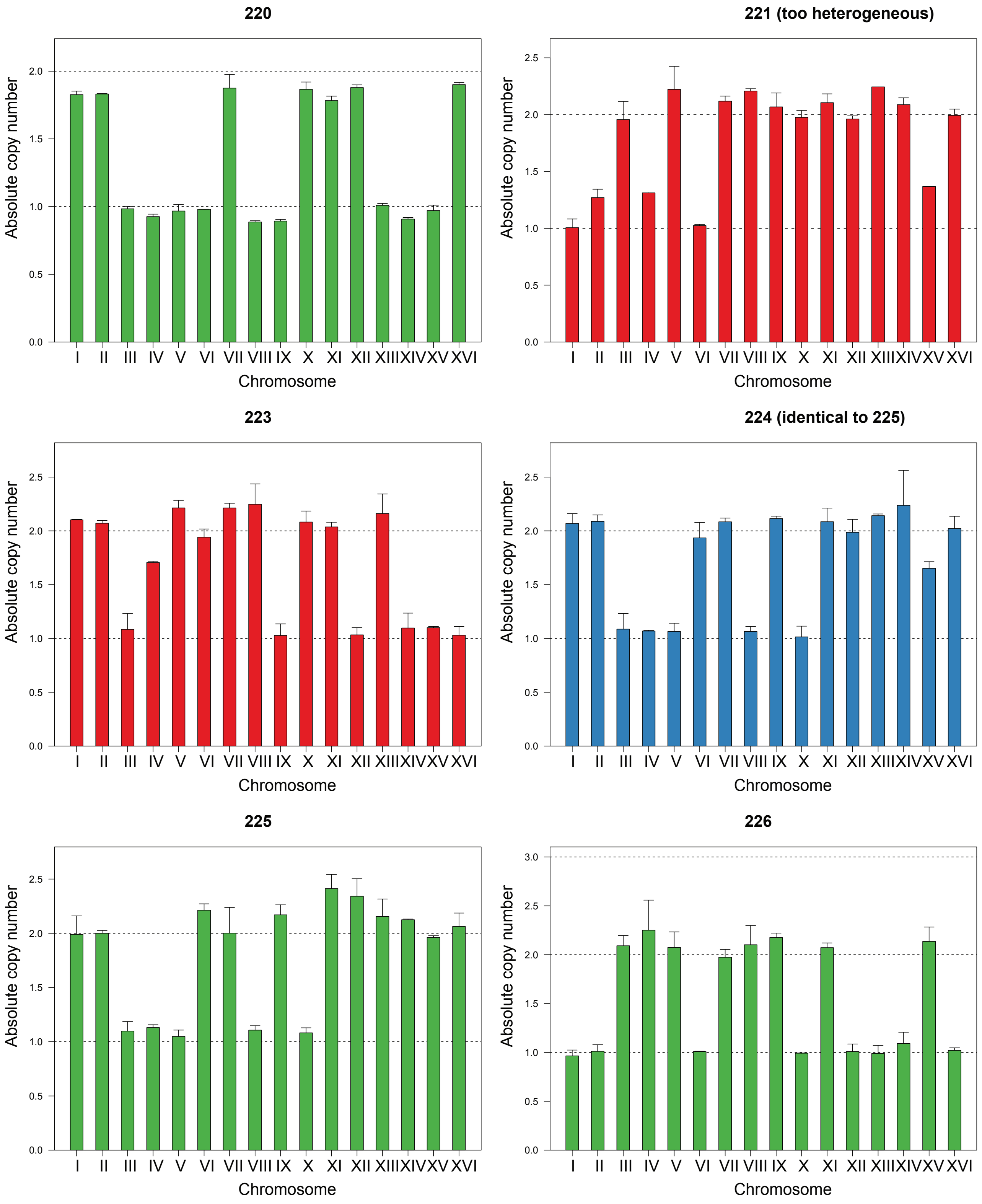

Figure S5 (page 3)

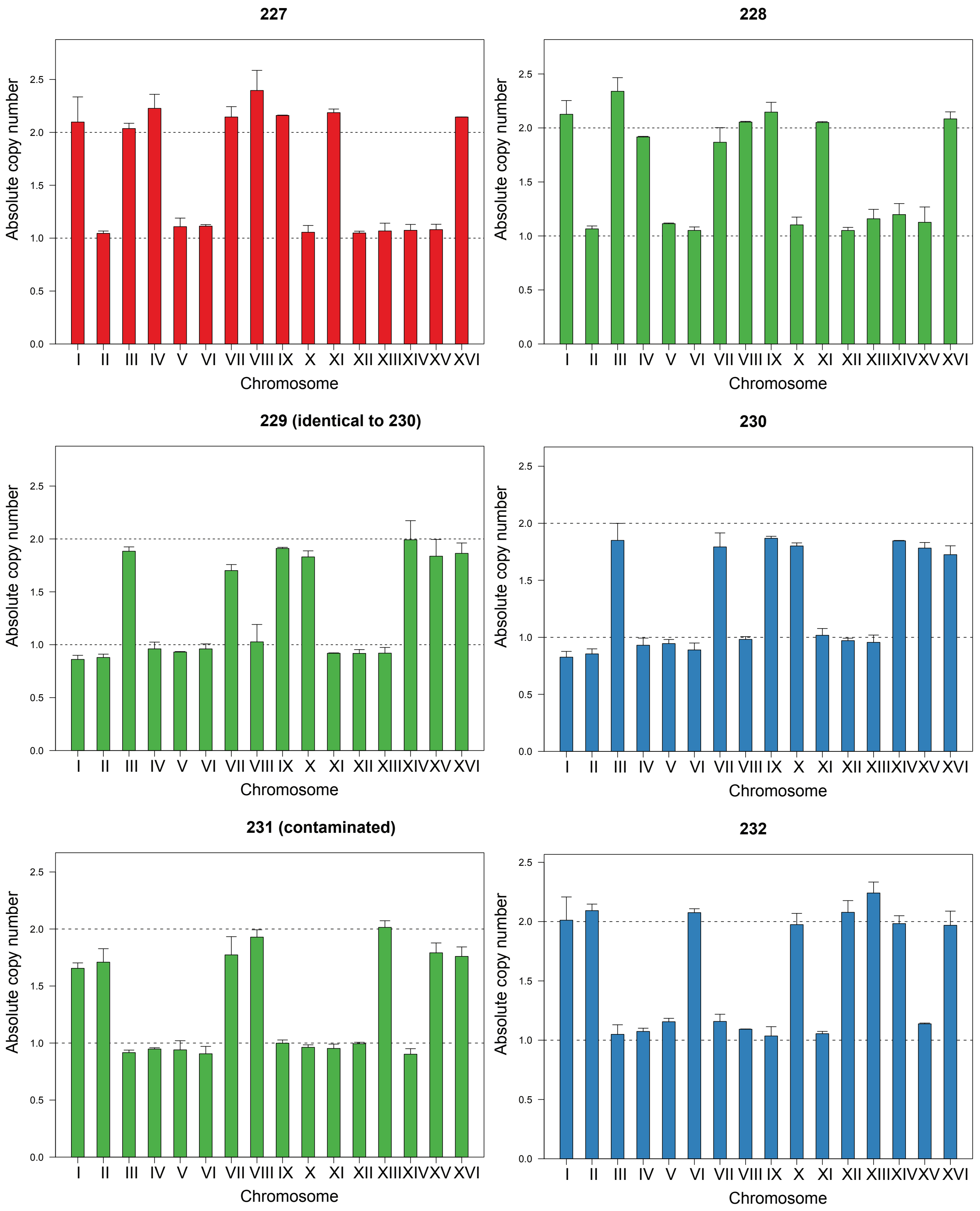

Figure S5 (page 4)

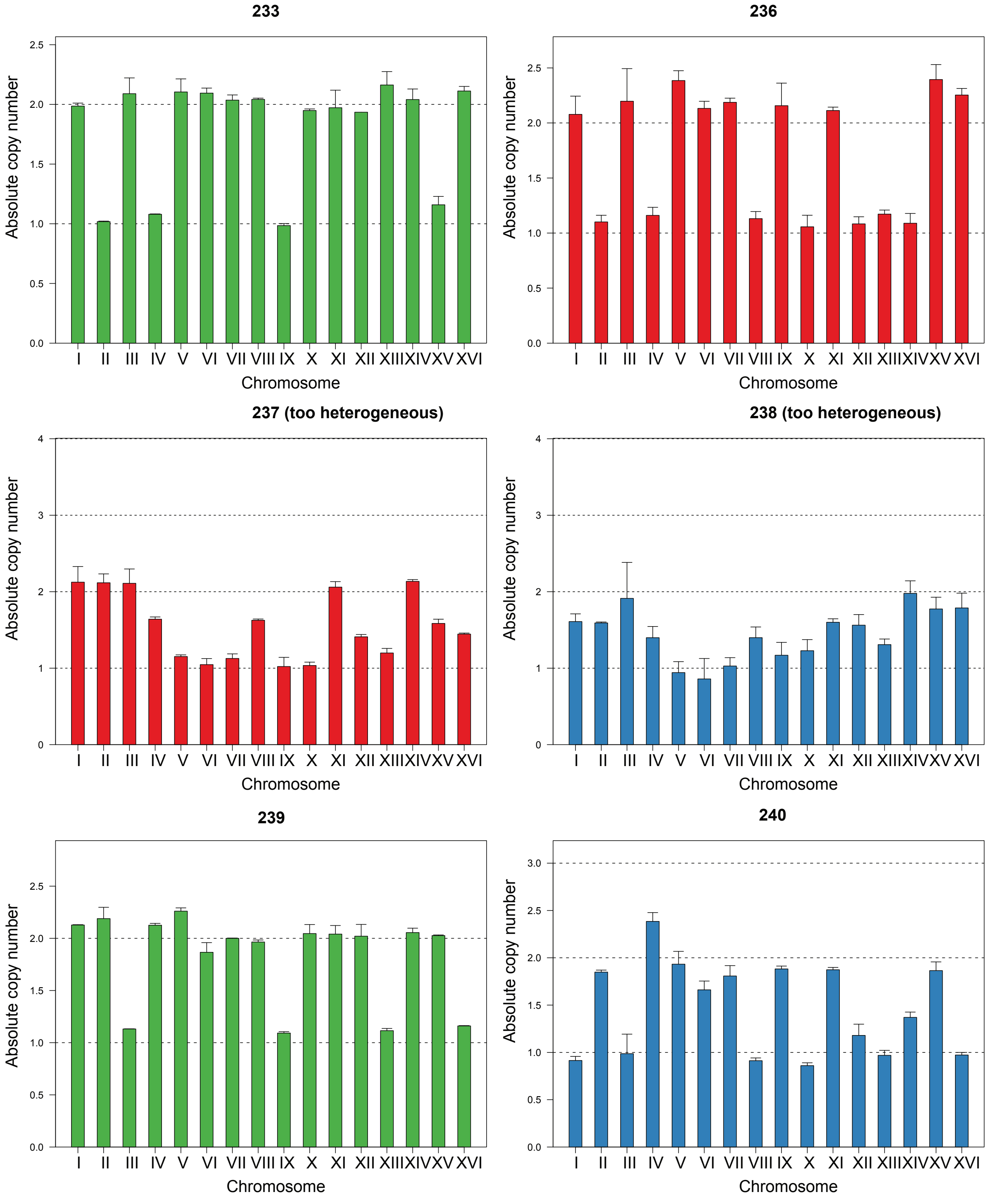

Figure S5 (page 5)

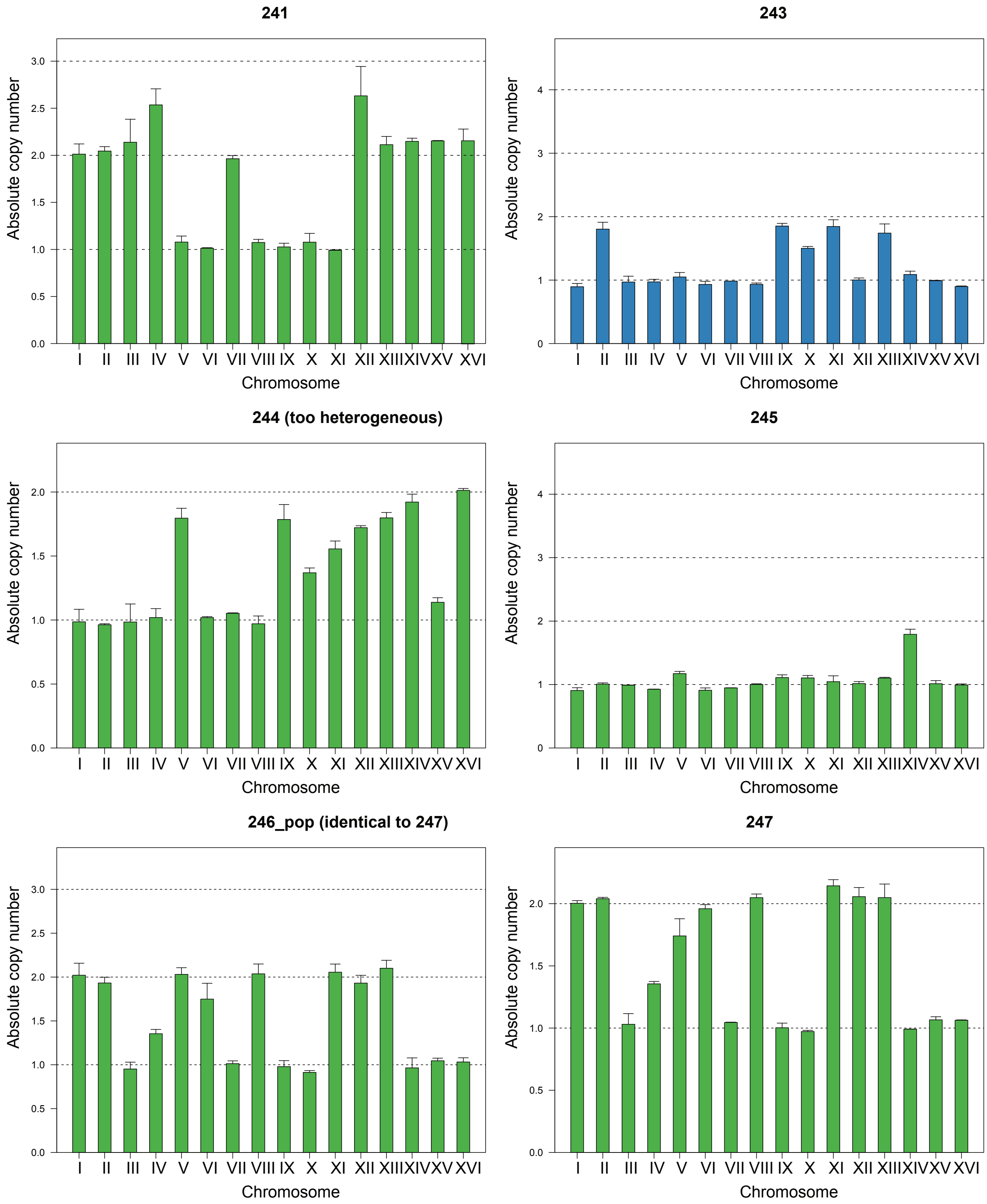

Figure S5 (page 6)

248 (too heterogeneous)

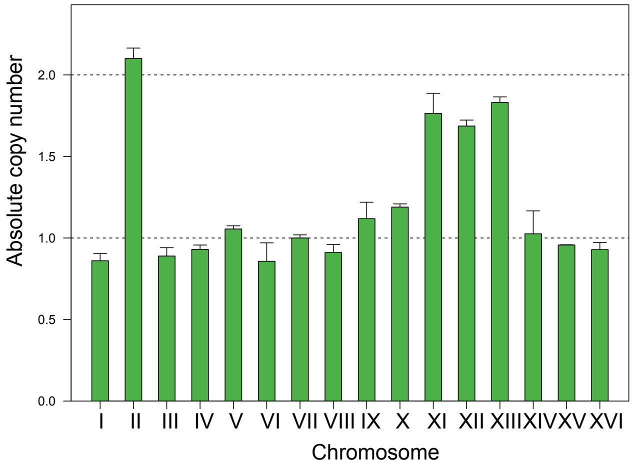

249

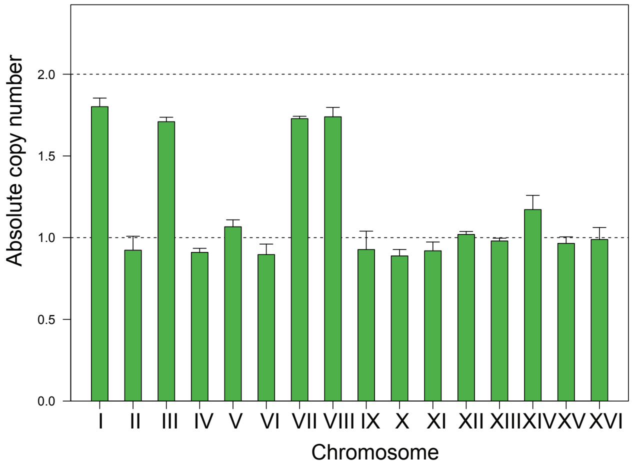

250 (very similar to 251)

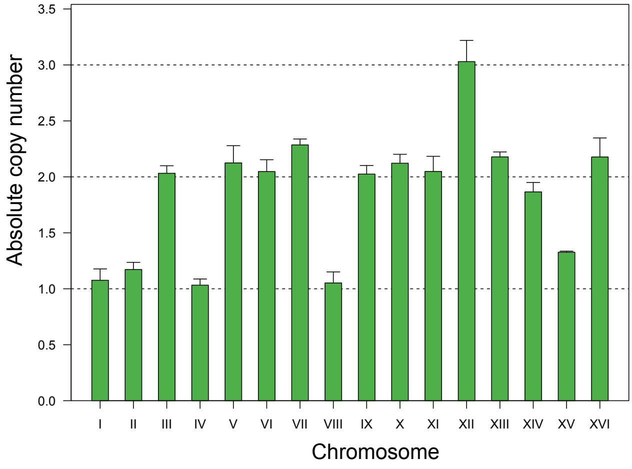

251

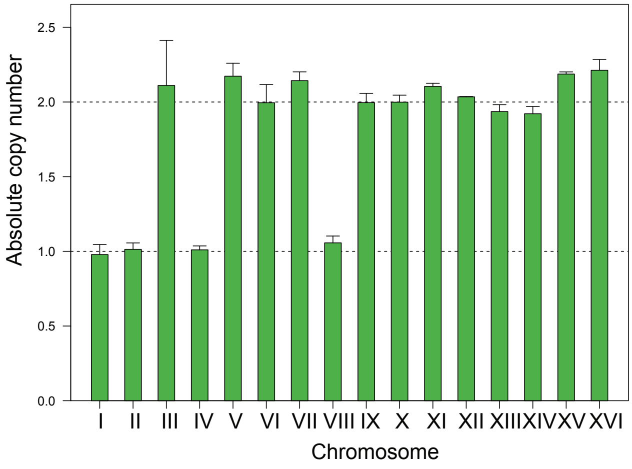

252

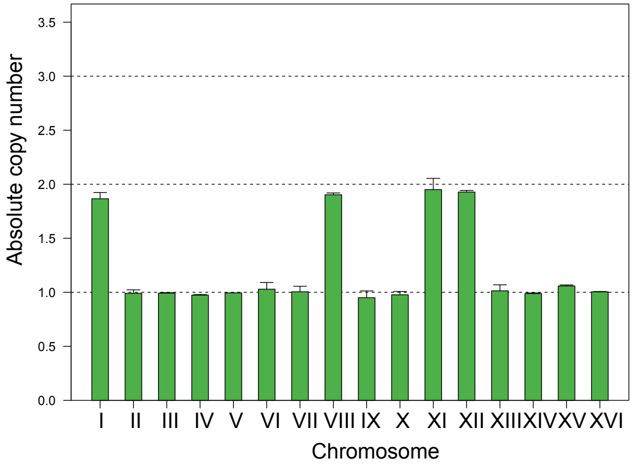

Figure S5 (page 7)
