# Supplementary material for: Karyotypic Determinants of Chromosome Instability in Aneuploid Budding Yeast
Source: PLoS Genet. 2012 May 17;8(5):e1002719. doi: 10.1371/journal.pgen.1002719 (PMC3355078; doi:10.1371/journal.pgen.1002719)
Supplement: Figure S7 — Karyotype information and karyotype networks of all 27 analyzed aneuploid strains. For all 27 analyzed aneuploid strains, karyotype makeups and reconstructed karyotype networks are shown. The number of CIN events used to qualitatively classify the aneuploid strains is shown on the right. See legend of Figure 2 for details on data presentation. Note that there exist two equally probable karyotype networks for strain 252, however in both cases the number of CIN events directly linked back to the original karyotype are the same hence its CIN classification is not affected. Alternative network is indicated by dashed lines. (PDF) [file pgen.1002719.s007.pdf]

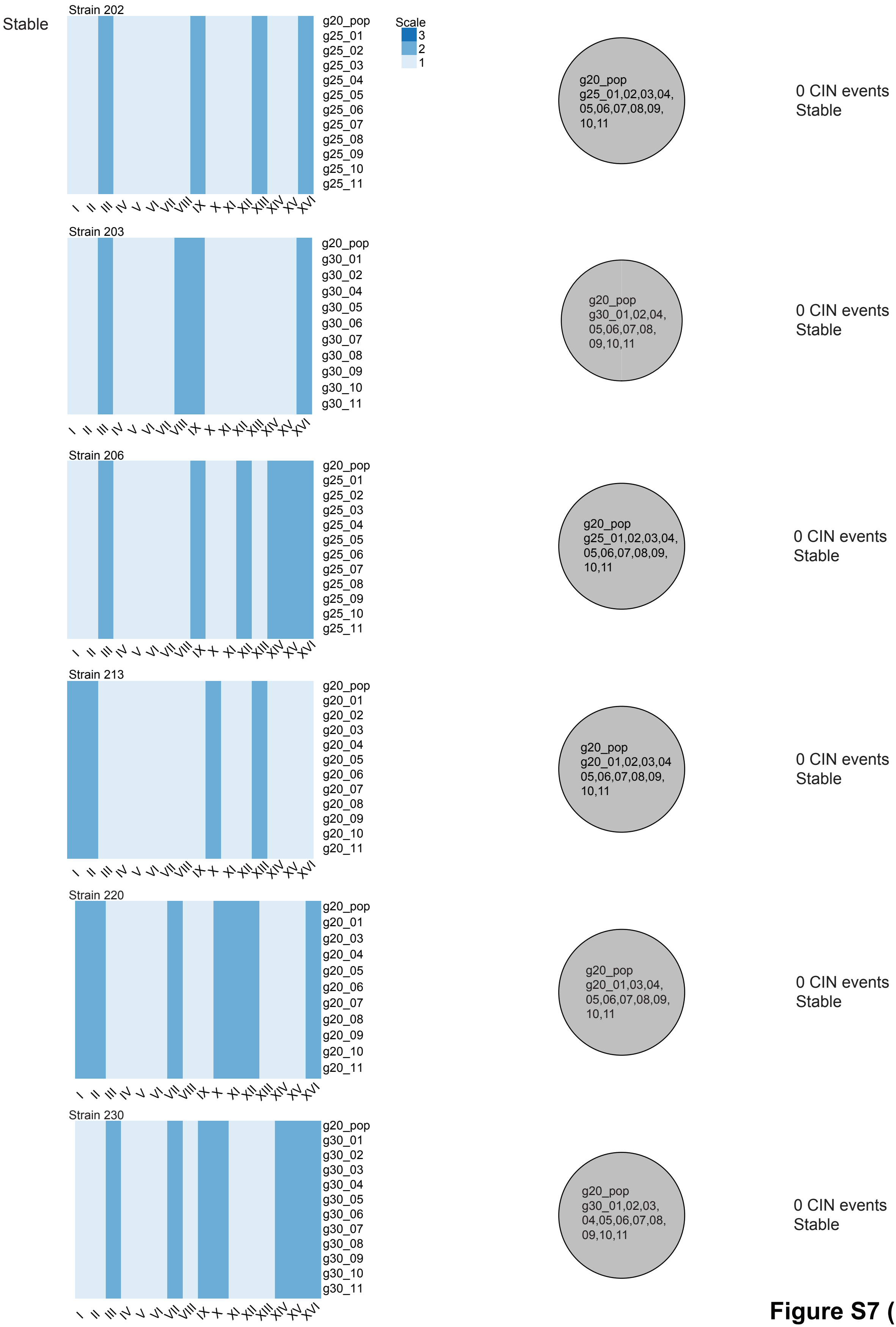

Figure S7 (Page 1)

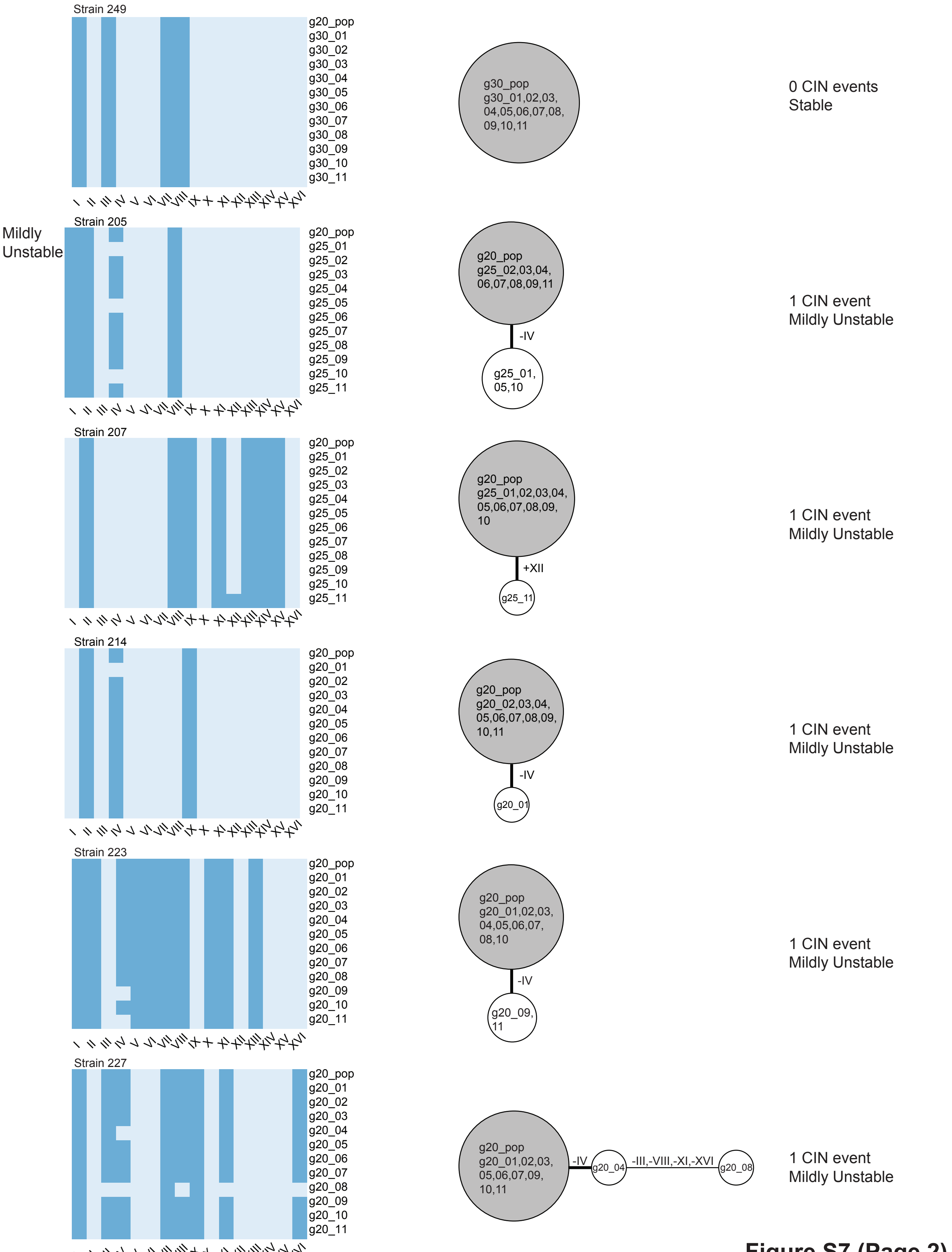

Figure S7 (Page 2)

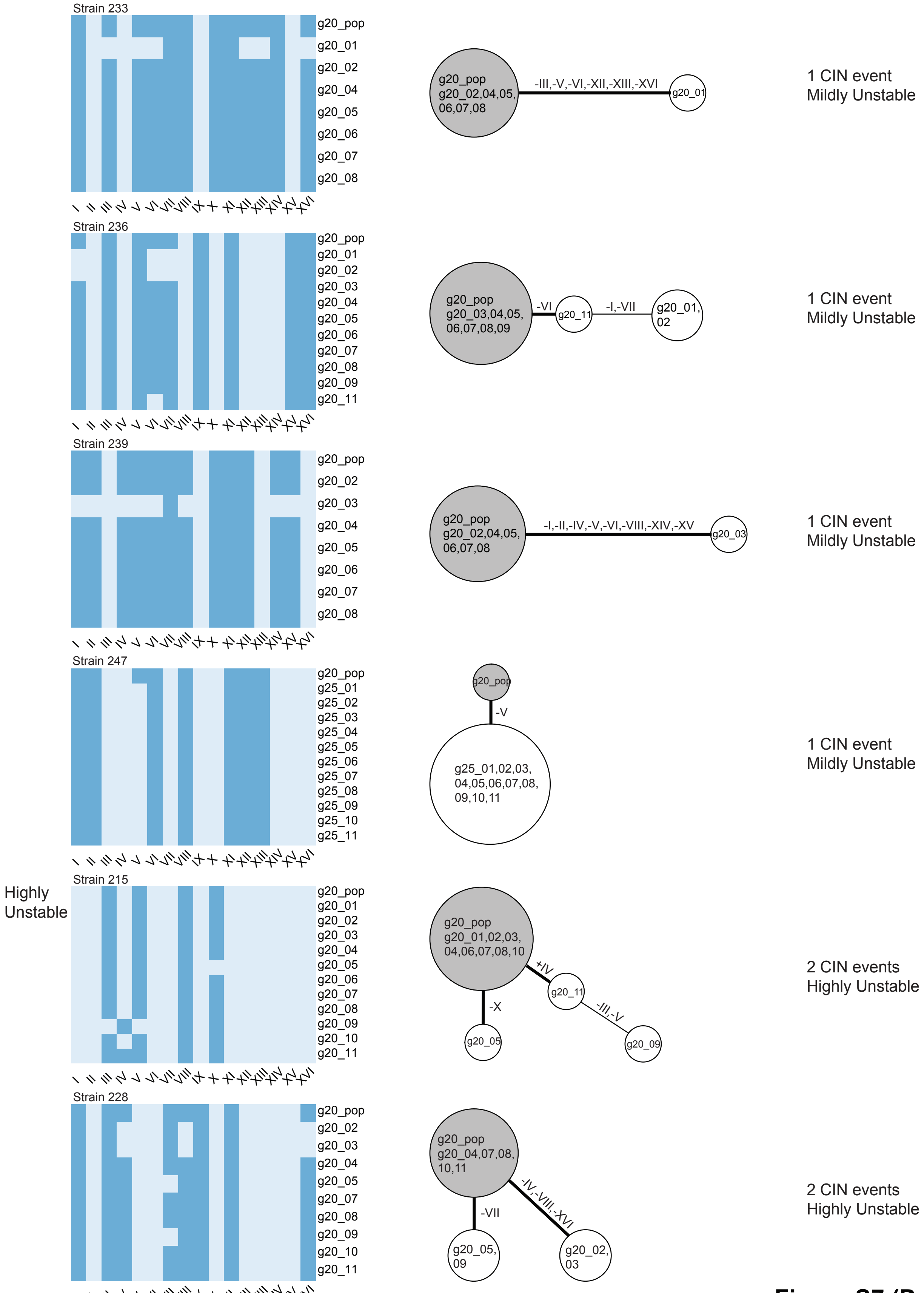

Figure S7 (Page 3)

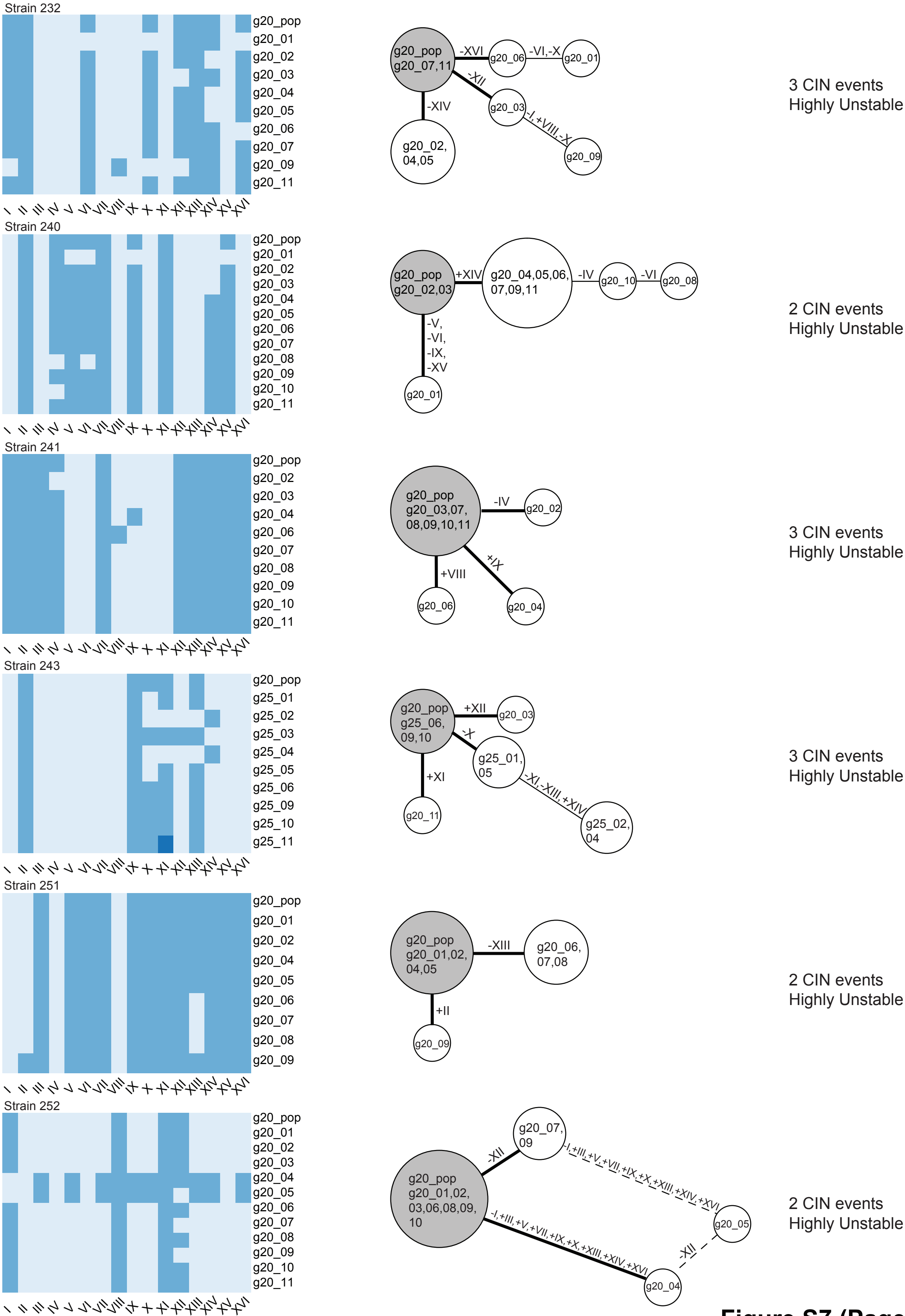

Figure S7 (Page 4)
